# Clusteroluminescence from Cluster Excitons in Small Heterocyclics Free of Aromatic Rings

**DOI:** 10.1002/advs.202004299

**Published:** 2021-02-11

**Authors:** Benzhao He, Jing Zhang, Jianyu Zhang, Haoke Zhang, Xiuying Wu, Xu Chen, Konnie H. S. Kei, Anjun Qin, Herman H. Y. Sung, Jacky W. Y. Lam, Ben Zhong Tang

**Affiliations:** ^1^ Department of Chemistry The Hong Kong University of Science and Technology Clear Water Bay Kowloon Hong Kong 999077 China; ^2^ Hong Kong Branch of Chinese National Engineering. Research Center for Tissue Restoration and Reconstruction Institute for Advanced Study Department of Chemical and Biological Engineering The Hong Kong University of Science and Technology Clear Water Bay Kowloon Hong Kong 999077 China; ^3^ HKUST‐Shenzhen Research Institute No. 9 Yuexing 1st RD, South Area, Hi‐tech Park Nanshan Shenzhen 518057 China; ^4^ Department of Polymer Science and Engineering Zhejiang University Xihu District Hangzhou 310027 China; ^5^ Center for Aggregation‐Induced Emission SCUT‐HKUST Joint Research Institute State Key Laboratory of Luminescent Materials and Devices South China University of Technology Guangzhou 510640 China; ^6^ AIE Institute Guangzhou Development District Huangpu Guangzhou 510530 China

**Keywords:** aggregation‐induced emission, clusteroluminescence, luminescent mechanism, maleimide, succinimide

## Abstract

The study of nonconventional luminescence is important for revealing the luminescence of natural systems and has gradually drawn the attention of researchers in recent years. However, the underlying mechanism is still inexplicable. Herein, the luminescence behavior of two series of simple, heteroatom‐containing small molecules without aromatic rings, i.e., maleimide and succinimide derivatives, are studied to gain further mechanistic insight into the nonconventional luminescence process. It has been unveiled that all the molecules exhibit bright and visible luminescence in concentrated solution and solid state and the formation of clusters is the root cause for such behaviors, which can effectively increase the possibility of both the nonradiative n–π* and favorable *π*–π* transitions and stabilize the excitons formed in the excited state. The distinctive luminescent phenomena and intriguing mechanism presented in this work will be significant for understanding the mechanism of clusteroluminescence and provide new strategies for the rational design of novel luminescent materials.

## Graphical abstract

Through systematic and comprehensive study on the luminescent performance of maleimide and succinimide derivatives, it has been unveiled that the formed clusters of these systems can effectively increase the possibility of the traditionally nonradiative n–π* transition and stabilize the excitons in the excited state to facilitate the emission.

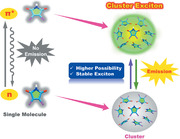

## 1 Introduction

**Figure 1:**
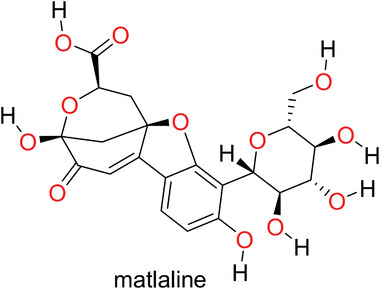
Structure of matlaline responsible for the fluorescence of lignum nephriticum.

The development of scientific exploration in humanity starts from studying the phenomena in nature, which provides continuous inspirations and lights the way for human progress. Regarding the fascinating luminescent phenomenon, its research began when a Spanish physician and botanist, called Nicolás Monardes, first discovered the peculiar luminescence of lignum nephriticum in 1565.^[^ ^1^ ^]^ It was recently reported that its unusual luminescence might stem from the nonconjugated matlaline (**Figure** **1**) and this mysterious luminescence is still attracting the interest of researchers.^[^ ^2^ ^]^ Inspired by such miraculous phenomena in nature, scientists began to artificially create luminescent compounds. Thanks to the development of organic chemistry, especially the progress of dye chemistry, many luminescent materials are available in various colors and diversity for different applications.^[^ ^3^ ^]^ On the other hand, many organic luminescent materials are *π*‐conjugated aromatic systems, in which their luminescence is often originated from the most favorable *π*–π* transition.^[^ ^3^, ^4^ ^]^ However, aromatic compounds, especially those with fused rings are usually toxic and are too stable to be biodegraded, which is harmed to the human health and pollutes the ecological environment.^[^ ^5^ ^]^ Therefore, the return to nature and the exploration and creation in accordance with the laws of nature are the fundamental laws for human beings to live in harmony with nature and keep moving forward.

It is noteworthy that deciphering and mimicking natural phenomena is fascinating but is also challenging. A particular challenge is that examples of conjugated aromatic systems, which are often called traditional luminescence paradigm, are limited in nature. In another aspect, many nonaromatic biomolecules such as proteins, enzymes, starch, cellulose, etc., that make up the organism are abundant in nature. And they are generally considered to be nonluminescent or weakly emissive due to the absence of intrinsic chromophores in their structures. However, it was gradually discovered that these nonaromatic structures containing heteroatoms are singularly luminescent in recent years but the involved mechanism is still inexplicable.^[^ ^6^ ^]^

As a matter of fact, the above mentioned natural systems can be regarded to show aggregation‐induced emission (AIE) as they exhibit only emission in the concentrated solution or solid state but emit no light in dilute solution.^[^ ^7^ ^]^ It has been revealed that the molecules of these natural compounds can interact with each other and form clusters driven by heteroatom‐involved intermolecular interactions to give rise to their unusual luminescence.^[^ ^8^ ^]^ Because these systems contain no any aromatic chromophores and their luminescence is derived from the formation of clusters and is mainly associated with n–π* transition, this special class of luminescence behaviors is termed as clusteroluminescence and the pertinent process is denoted as “clusterization‐triggered emission.”^[^ ^8^, ^9^ ^]^ Apart from these natural biopolymers, many new and artificially synthetic clusteroluminogens emerge and are documented in recent years, including macromolecules, supramolecular assemblies, nonconjugated polymer dots, siloxanes, metal clusters, and small molecular compounds.^[^ ^8^, ^9^, ^10^ ^]^

It is evident that such natural biomolecules and most of the synthetic systems showing clusteroluminescence are intricate, which brings great difficulties to study their luminescence mechanisms. In another aspect, maleimide and succinimide are generally known as effective fluorescence quenchers originated from their low‐lying n–π* state and they are widely used to construct turn‐on probes for the detection of thiols due to the distinct luminescence of the resulting substituted products.^[^ ^11^ ^]^ Indeed, some researchers found that alkyl or aryl substituted maleimide and succinimide derivatives exhibited impressive luminescence in the condensed state.^[^ ^12^ ^]^ However, the inherent mechanism of their luminescence is still obscure although it has been tentatively uncovered in few previous works.^[^ ^12b,d^ ^]^ Therefore, the thorough disclosure of their luminescence mechanisms is of great significance for the design of related probes. Additionally, it should be mentioned that we recently discovered that these compounds all exhibit typical clusteroluminescent characteristics in the condensed state. Therefore, we unambiguously selected these simple heteroatom‐containing maleimide and succinimide derivatives as models and conducted detailed investigation on their luminescence properties to gain further mechanistic insight into the clusteroluminescence and to provide inspirations for the design of new maleimide‐ and succinimide‐based turn‐on probes.

**Scheme 1:**
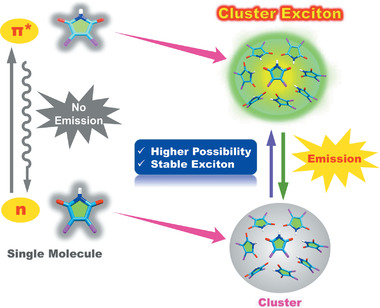
Schematic illustration of cluster exciton facilitated emission from n–π* transition.

Herein, we elaborately introduced different alkyl thiols to both maleimide and succinimide heterocyclic frameworks and designed two series of compounds in this work. We carried out a systematic and comprehensive study on their luminescent performance and proposed a new mechanism for their luminescence. It turns out that these compounds all exhibit typical clusteroluminescence and their unique luminescence is intrinsically derived from the cluster excitons. More importantly, for the first time, it has been unveiled that the formed clusters of these nonconjugated systems can effectively increase the possibility of the traditionally nonradiative n–π* transition and stabilize the excitons in the excited state to prominently facilitate the emission (**Scheme** **1**).

## 2 Results and Discussion

Maleimide (MI) and succinimide (SI) are commercially available compounds. The other four target compounds, 2‐ethylthio maleimide (ETMI), 2,3‐diethylthio maleimide (DETMI), 2‐ethylthio succinimide (ETSI), and 2,3‐diethylthio succinimide (DETSI), were prepared by simple one‐step reaction (Scheme S1, see the Supporting Information for details). All of the target products were characterized using NMR and high‐resolution mass spectroscopy with satisfactory results (Figures S1–S24, Supporting Information). Additionally, their molecular structures were further confirmed by single‐crystal X‐ray diffraction (details see below), and the relevant data were provided in Table S1 in the Supporting Information. It is noteworthy to mention that Yan et al. reported the strong green fluorescence of dithiosuccinimides (DTS) in 2015 and we also observed the consistent emission for the product purified by silica gel column chromatography.^[^ ^12b^ ^]^ However, when we further analyzed its purity, we found that it actually contains a small amount of impurity. Accordingly, further purification by high performance liquid chromatography (HPLC) was carried out and we surprisingly found that the ultimate product of DTS only emitted weak cyan light. This result indicates that the strong green fluorescence we initially observed is originated form the impurity. Therefore, we collected the samples purified by HPLC and then caried out the following photophysical experiments to exclude the influence of impurity.

**Figure 2:**
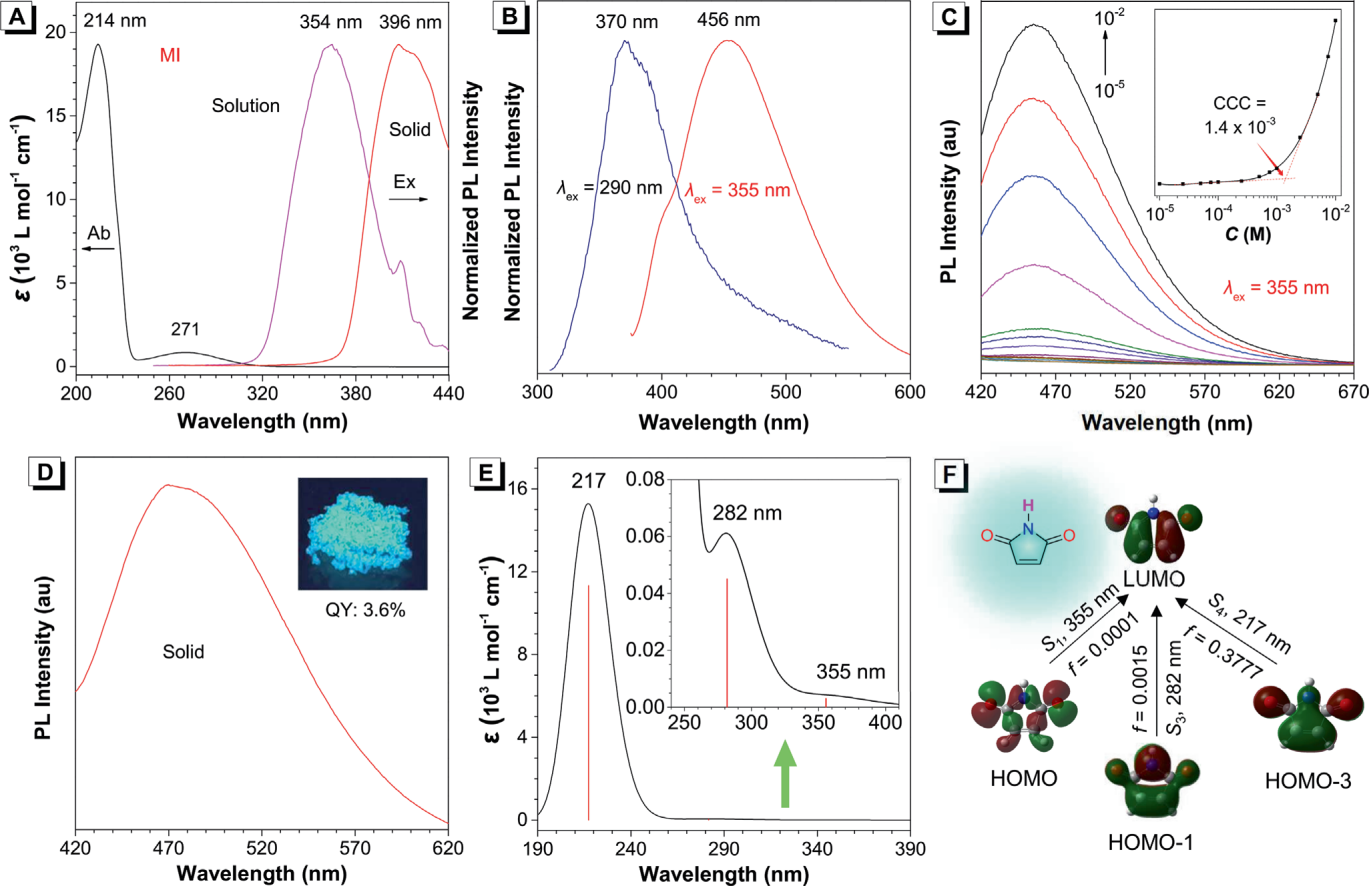
A) UV–vis absorption (*c* = 1 × 10^−4^ m), and excitation spectra of MI in acetonitrile (MeCN) solution (magenta line, *λ*_em_ = 456 nm, *c* = 1 × 10^−2^ m) and solid state (red line). B) Photoluminescence (PL) spectra of MI in MeCN at different excitation wavelengths (*c* = 1 × 10^−2^ m). C) PL spectra of MI in MeCN with different concentration (*λ*_ex_ = 355 nm). Inset: plot of PL intensity of MI versus concentration. D) PL spectra of MI in the solid state (*λ*_ex_ = 396 nm). Inset: photo taken under 365 nm UV irradiation. E) UV–vis absorption and oscillator strength of MI, calculated by using B3LYP/6‐31G* /CPCM/MeCN method with Gaussian 09 program. F) Spin orbitals involved in the major electronical excitations in MI, calculated by using B3LYP/6‐31G* /CPCM/MeCN method with Gaussian 09 program. Abbreviation: CCC = critical cluster concentration, QY = quantum yield.

First, their photophysical properties were systematically studied. As showed in **Figure** **2**A, MI exhibited a strong absorption band at 214 nm, and a week one at 271 nm. When it was excited with its maximum absorption wavelength of 270 nm, a weak emission at 370 nm was observed for its condensed solution (Figure 2B), which should be ascribed to the intrinsic luminescence of MI ring. Interestingly, a remarkably red‐shifted emission located at 456 nm could be observed when a longer excitation wavelength (*λ*_ex_) of 355 nm was applied (Figure 2B). Based on this phenomenon, we presumed that this unique emission was probably originated from the formation of clusters in the concentrated solution.^[^ ^13^ ^]^ To confirm this hypothesis, the luminescence behaviors of MI at different concentrations, from 1 × 10^−5^ m to 1 × 10^−2^ m, were studied under 355 nm excitation. As illustrated in Figure 2C, the dilute solution of MI was nonemissive. However, with the concentration increasing, its photoluminescence (PL) intensity gradually increased very slightly at low concentration, but then enhanced very sharply when the concentration reached higher than 10^−3^ m, which is attributed to the typical characteristics of clusterization‐triggered emission (CTE).^[^ ^9^ ^]^ Theoretically, it is difficult to form clusters at low concentration and the PL intensity shows no obvious change because it is hard for molecules to interact with each other. When the concentration was increased to a critical point, the clusters were formed via sufficient intermolecular interactions, which leads to a remarkable enhancement in PL intensity. Herein, we defined this critical point as critical cluster concentration (CCC), and the CCC value of MI was determined as 1.4 × 10^−3^ m (Figure 2C, inset and Table S2, Supporting Information). Actually, the results of dynamic light scattering (DLS) measurement further verified the formation of clusters. As shown in Figure S25 (Supporting Information), large size of clusters could be detected by DLS when the concentration of MI exceeded CCC, but not when it was lower than the CCC point. Additionally, it was observed that the size of the clusters decreased when increasing the concentration from 2.5 × 10^−3^ m to 1 × 10^−2^ m, indicating that much tighter clusters may form to result in brighter emission.

Solid state PL spectrum of MI further verified the formation of clusters. As demonstrated in Figure 2D, the maximum emission wavelength of MI in solid state showed a prominent redshift compared with that of its solution state, and exhibited a much higher quantum yield (QY) of 3.6% (Table S2, Supporting Information). The excitation spectrum of MI powder showed that the maximum excitation wavelength located at 396 nm, indicating an obvious redshift in comparison with its solution state. Based on these observations, we proposed that the red‐shifted emission and excitation in the solid state were derived from the newly formed cluster species in the condensed state, which is completely distinguished from its monomolecular state in dilute solution.^[^ ^9^, ^13^ ^]^

To validate the above proposal, density functional theory (DFT) calculations based on MI molecule were carried out (details see the Supporting Information). According to the calculated results (Figure 2E,F; Table S3, Supporting Information), the absorption peaks of MI at 214 and 271 nm in the experimental UV spectra have been well duplicated and both can be attributed to the *π*–π* transitions localized on the central five‐membered ring. It is noteworthy that there is a weak absorption at 355 nm which completely matches the experimental excitation wavelength in solution state (Figure 2A). The calculation results suggest that this peak is corresponding to the forbidden n–π* transition from carbonyl group to the central ring (Table S3, Supporting Information) with small oscillator strength (*f*) value of 0.0001, which is the reason why this peak was not detected in the UV spectra and no obvious emission was obtained in the dilute solution when excited with 355 nm.^[^ ^14^ ^]^ Considering that its remarkable luminescence appeared in high concentrations, the absorption spectra of MI in different concentration were collected and a gradually enhanced shoulder peak at around 340–360 nm was observed with the increasing of concentration (Figure S26, Supporting Information). Therefore, taken all the experimental and calculated results together, we reasoned that the formation of clusters in high concentrations would dramatically promote the possibility of n–π* transition and the resultant cluster exciton in the excited state should be much more stable, which ultimately results in the distinctive fluorescence of such a small molecule.^[^ ^15^ ^]^

**Figure 3:**
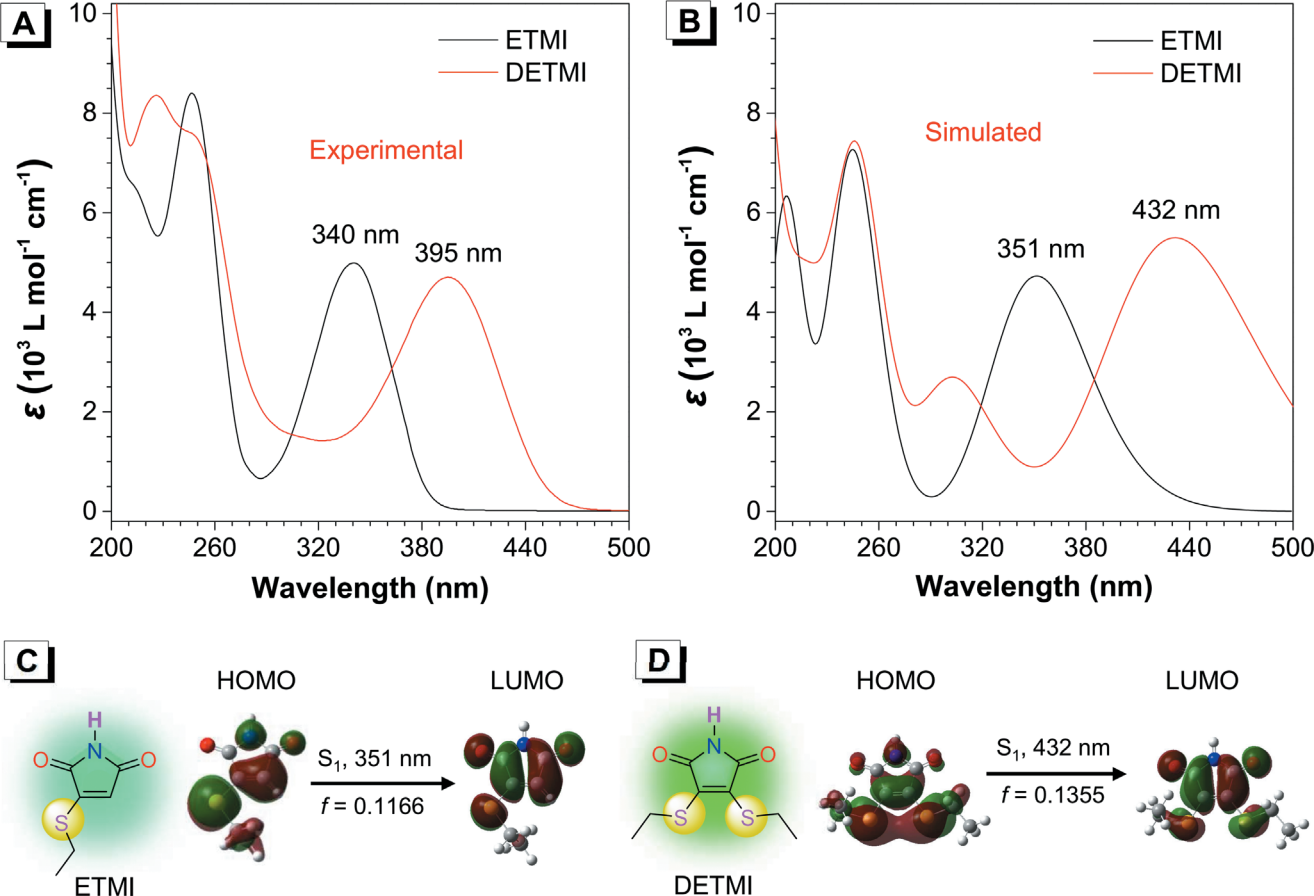
A) Experimental UV–vis absorption spectra of ETMI and DETMI in MeCN (*c* = 1 × 10^−4^ m), B) Calculated UV/vis absorption spectra of ETMI and DETMI, by using B3LYP/6‐31G*/CPCM/MeCN method with Gaussian 09 program. C,D) Structures and energy levels of HOMO and LUMO of C) ETMI and D) DETMI calculated by using B3LYP/6‐31G*/CPCM/MeCN method with Gaussian 09 program. Purple: nitrogen atom; yellow: sulfur atom; red: oxygen atom; cyan: carbon atom; white: hydrogen atom.

Considering that heteroatoms in nonconventional luminescent systems play critical roles in CTE, sulfur atoms were further introduced into MI molecule to examine their effect on clusteroluminescence. By introducing one ethanethiol and two ethanethiol groups into MI framework, two target molecules of ETMI and DETMI were obtained, respectively (**Figure** **3**). Surprisingly, in sharp contrast to MI, these two compounds both exhibited strong absorptions at longer‐wavelength with maximum absorption (*λ*_abs_) at 340 and 395 nm, respectively (Figure 3A). The calculated absorption spectra are almost the same as the experimental ones, except for a slight redshift, with *λ*_abs_ at 351 and 432 nm, respectively (Figure 3B). Analysis of their frontier molecular orbitals suggested that the nature of S_1_ changed from n–π* transition in MI to *π*–π* transitions in both ETMI and DETMI with increased *f* (Figure 3C,D). Meanwhile, in the highest occupied molecular orbital (HOMO), the electrons are mainly localized on the sulfur and C=C double bond and then delocalized on the central five‐membered rings at the lowest unoccupied molecular orbital (LUMO). All these results indicated that the introduction of S atoms can alter the nature of the transition and afford a transformation from low‐probability dark n–π* state to more favorable bright *π*–π* state (Table S3, Supporting Information),^[^ ^14a^ ^]^ which is considered to facilitate clusteroluminescence.

**Figure 4:**
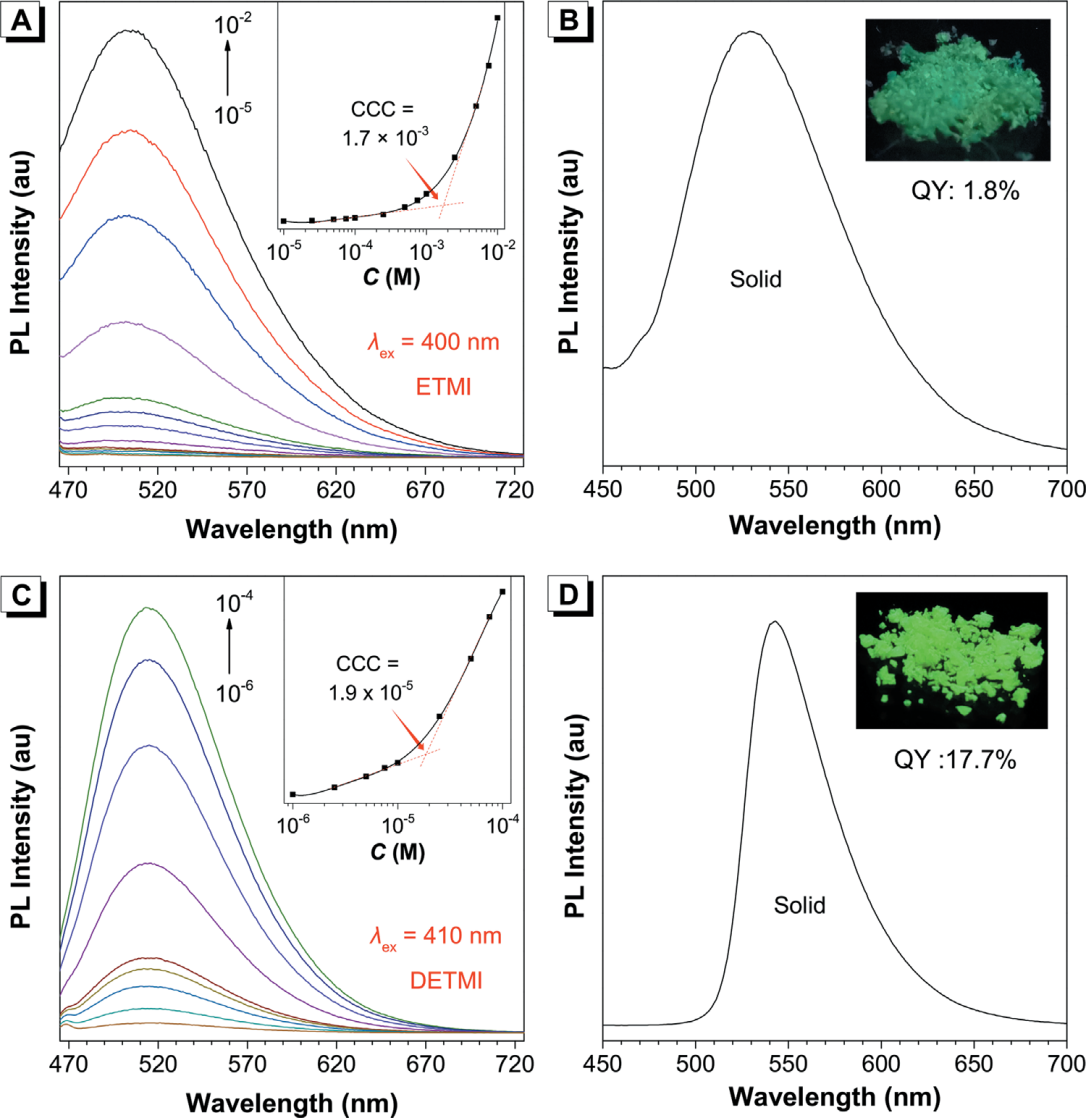
A) PL spectra of ETMI in MeCN with different concentration (*λ*_ex_ = 400 nm). B) PL spectra of ETMI in solid state (*λ*_ex_ = 446 nm). C) PL spectra of DETMI in MeCN with different concentration (*λ*_ex_ = 410 nm). D) PL spectra of DETMI in solid state (*λ*_ex_ = 465 nm). Insets: plot of PL intensity versus concentration and photos of ETMI and DETMI in the solid state taken under 365 nm UV lamp. Abbreviation: QY = quantum yield.

The luminescent properties of ETMI and DETMI in solution and solid state were further characterized. As shown in **Figure** **4**A,C, the PL evolutions of ETMI and DETMI in acetonitrile solution with the increase of concentration were very similar to that observed in MI and their CCC values were determined as 1.7 × 10^−3^ and 1.9 × 10^−5^ m, respectively. By comparing to MI, the maximum emission peaks (*λ*_em_) of ETMI and DETMI in solution state showed obvious redshift to 500 and 513 nm, respectively, and *λ*_em_ in the solid state exhibited a further shift to 529 and 540 nm, respectively (Figure 4B,D). The above results suggested that the introduction of S atoms could dramatically facilitate the clusteroluminescence via altering the nature of S_1_ from n–π* transition to efficient *π*–π* transition. Thus, the emission of DETMI is much more prominent showing a relatively much lower CCC value and its solid‐state QY value reaches up to 17.7% (Figure 4C,D, insets).

**Figure 5:**
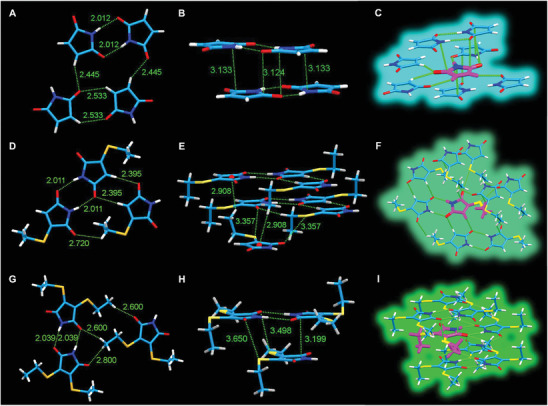
A,D,G) Intermolecular hydrogen bonding and B,E,H) D···A, *π*···*π*, and O···S interactions of MI, ETMI, and DETMI, respectively. C,F,I) Clusters formed from MI, ETMI, and DETMI molecules, respectively.

Fortunately, single crystal structures of MI, ETMI, and DETMI were all obtained, which provided more solid evidence for the CTE mechanism as proposed above. As illustrated in Figure 5, there are multiple strong hydrogen bonds (**Figure** **5**A) and *π*–*π* interactions (Figure 5B) among the neighboring MI molecules. Because of these interactions, the molecules could be fully rigidified and form a stable cluster (Figure 5C), which acts as an ideal luminescent unit. Regarding ETMI, there are C–H···*π* interactions and unique O···S interactions due to the introduction of one sulfur atom into the structure (Figure 5D,E),^[^ ^16^ ^]^ which results in the formation of stable cluster in ETMI (Figure 5F). Impressively, strong intramolecular S···S interaction exists in DETMI molecule because of the introduction of two S atoms (Figure S27, Supporting Information), thus rendering the molecule more rigid and that is the reason why strong emission could be observed even in its dilute solution.^[^ ^10^, ^17^ ^]^ Meanwhile, intermolecular multiple hydrogen bonds, *π*–*π* interactions and unique O···S interactions in DETMI also played important roles in the formation of cluster structure (Figure 5G–I).

Based on the abovementioned crystal data as well as the experimental and calculated results, it is easy to conclude that these aromatic‐free maleimide‐based structures could form clusters in concentrated solution or solid states. The formation of clusters can efficiently increase the possibility of both the nonradiative n–π* and radiative *π*–π* transitions and stabilize the excitons formed in the excited state, which gives rise to the efficient luminescence in their clustering states.

**Figure 6:**
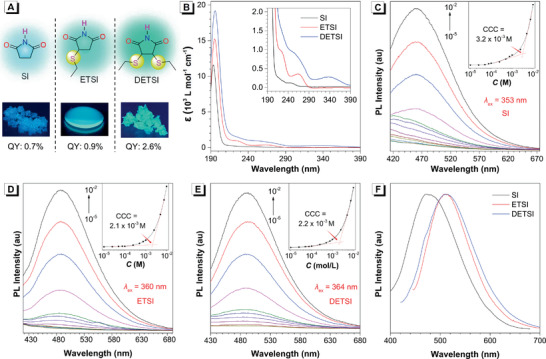
A) Structures of SI, ETSI, and DETSI and the corresponding photos of their solid states taken under 365 nm UV light. B) UV–vis absorption of SI, ETSI, and DETSI in MeCN (*c* = 1 × 10^−4^ m). C) PL spectra of SI in MeCN with different concentration (*λ*_ex_ = 353 nm). D) PL spectra of ETSI in MeCN with different concentration (*λ*_ex_ = 360 nm). E) PL spectra of DETSI in MeCN with different concentration (*λ*_ex_ = 364 nm). E) PL spectra of SI, ETSI and DETSI in solid state (*λ*_ex_ = 375, 400, and 410 nm, respectively). Abbreviation: QY = quantum yield.

Therefore, to check whether the clusterization is a general approach to facilitate the emission, a series of flexible succinimide derivatives (SI, ETSI, and DETSI) were prepared in which the C=C double bond was replaced by single bonds (**Figure** **6**A). The purpose was to obtain high ratio of n–π* character and verify the feasibility of CTE approach to the luminescence of n–π* involved dark state (Table S3, Supporting Information). As predicted by calculations (Figure S28 and Table S3, Supporting Information), the main absorption bands below 300 nm of these three compounds were weakened and blue‐shifted in comparison with the corresponding conjugated MI derivatives, which are consistent with the experimental results (Figure 6B). Additionally, as we expected, the introduction of S atoms leads to dominant n–π* transitions in these three compounds (Figure S29 and Table S3, Supporting Information).

The PL spectra of these three compounds suggested that there is almost no luminescence in their dilute solution states. When increasing their respective concentrations, the PL intensity of them all showed slight enhancement initially, but then intensified dramatically when the concentration reached above their respective CCC (Figure 6C–E). These evolutions were similar to those observed in the maleimide‐based systems, also indicating their CTE characteristics. It is worth noting that the CCC values of succinimide derivatives are larger than that of maleimide‐based series, which is caused by their more flexible structures that a higher clusterization concentration is required to rigidify and stabilize the cluster. Meanwhile, their solids were more emissive due to the formation of condensed clusters and their QY showed a gradual increase with the increased number of S atoms (Figure 6A). The maximum emission wavelength of SI, ETSI, and DETSI in solid state are located at 480, 513, and 511 nm, respectively, indicating a prominent redshift compared with that of their respective solution states (Figure 6F). Therefore, these results well answered our question addressed above: the clusterization is indeed a general and efficient approach to achieving the luminescence of n–π* involved dark state.

**Figure 7:**
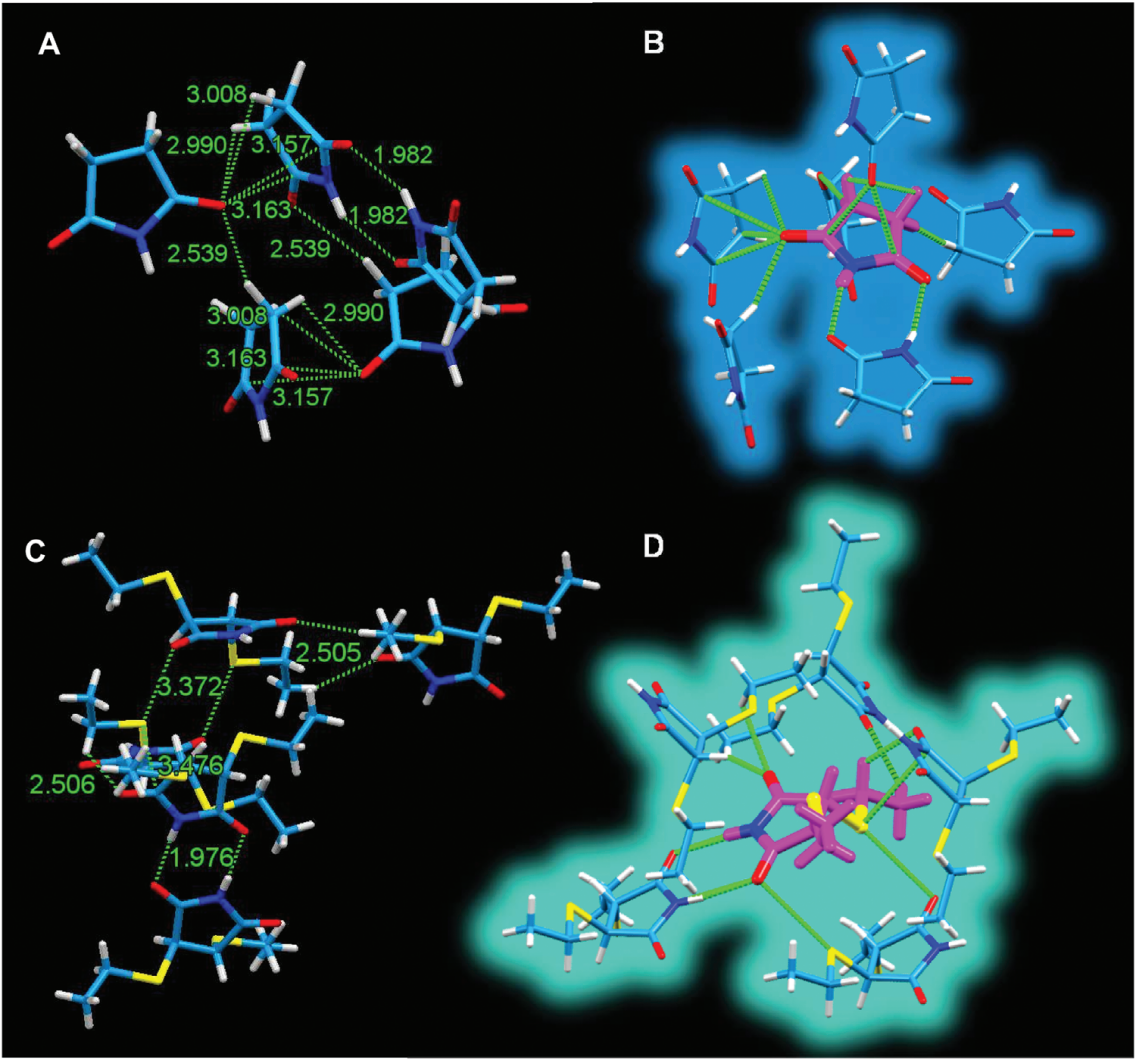
A) Intermolecular hydrogen bonding and C···O interaction in SI. C) Intermolecular hydrogen bonding, C···S and O···S interactions in DETSI. B,D) Cluster formed from SI and DETSI molecules, respectively.

Crystal structures of SI and DETSI demonstrated the formation of clusters (**Figure** **7**). As shown in Figure 7A,B, the SI molecules are flexible and form a spatial cluster structure through hydrogen bond and electrostatic C···O interactions. For DETSI, the introduction of two S heteroatoms provided additional C···S and O···S interactions to help form a more rigid cluster (Figure 7C,D). Since ETSI is liquid at room temperature, it is unable to grow its single crystal. Accordingly, it can be summarized that the clusterization is indeed a general and effective approach to achieving unique luminescence for the unconventional systems even the nonradiative n–π* involved systems.

## 3 Conclusions

In this work, we designed and synthesized maleimide (MI) and succinimide (SI) derivatives without aromatic rings and systematically studied their luminescence behaviors. We found that all the compounds exhibit unique luminescence in concentrated solution and solid state due to the cluster formation. Specifically, in the dilute solution and monodispersed state, MIs and SIs are nonemissive because of the involved almost forbidden n–π* transitions. However, in concentrated solution or solid state, they are emissive due to the formation of clusters, which can effectively increase the possibility of n–π* transitions and stabilize the excitons formed in the excited state. Additionally, in the maleimide‐based systems, the introduction of ethanethiol groups could convert n–π* into *π*–π* transitions and introduce intramolecular S···S interaction to dramatically help enhancing the luminescence. In the succinimide‐containing systems, the introduction of S atoms could lead to a dominant n–π* transition and the clusterization process could also efficiently boost their emission. Therefore, our present results demonstrate that the clusterization is a general and effective approach to achieve unique luminescence of unconventional systems. Therefore, this work is anticipated to provide new insight into clusteroluminescence and open a new way for the design of novel clusteroluminogens.

## Conflict of Interest

The authors declare no conflict of interest.

## Supporting information

Supporting Information
