# Supplementary material for: Clusteroluminescence from Cluster Excitons in Small Heterocyclics Free of Aromatic Rings
Source: Adv Sci (Weinh). 2021 Feb 11;8(7):2004299. doi: 10.1002/advs.202004299 (PMC8025018; doi:10.1002/advs.202004299)
Supplement: Supplementary file 1 — Supporting Information [file ADVS-8-2004299-s001.pdf]

WILEY-VCH

© 2020 Wiley-VCH GmbH

ADVANCED SCIENCE
Open Access

Supporting Information

for *Adv. Sci.*, DOI: 10.1002/advs.202004299

Clusteroluminescence from Cluster Excitons in Small Heterocyclics Free of Aromatic Rings

Benzhao He, Jing Zhang, Jianyu Zhang, Haoke Zhang, Xiuying Wu, Xu Chen, Konnie H. S. Kei, Anjun Qin, Herman H. Y. Sung, Jacky W. Y. Lam, and Ben Zhong Tang**

Supporting Information

Clusteroluminescence from Cluster Excitons in Small Heterocyclics Free of Aromatic Rings

Benzhao He, Jing Zhang, Jianyu Zhang, Haoke Zhang, Xiuying Wu, Xu Chen, Konnie H. S. Kei, Anjun Qin, Herman H. Y. Sung, Jacky W. Y. Lam, and Ben Zhong Tang**

Dr. B. He, Dr. J. Zhang, J. Zhang, K. H. S. Kei, Prof. H. H. Y. Sung, Dr. J. W. Y. Lam, Prof. B. Z. Tang
Department of Chemistry, The Hong Kong University of Science and Technology, Clear Water Bay, Kowloon, Hong Kong, China
E-mail: tangbenz@ust.hk

Dr. B. He, Dr. J. Zhang, J. Zhang, K. H. S. Kei, Dr. J. W. Y. Lam, Prof. B. Z. Tang
Hong Kong Branch of Chinese National Engineering. Research Center for Tissue Restoration and Reconstruction, Institute for Advanced Study, Department of Chemical and Biological Engineering, The Hong Kong University of Science and Technology, Clear Water Bay, Kowloon, Hong Kong, China

Dr. B. He, Dr. J. W. Y. Lam, Prof. B. Z. Tang
HKUST-Shenzhen Research Institute, No. 9 Yuexing 1st RD, South Area, Hi-tech Park, Nanshan, Shenzhen, 518057, China

Dr. H. Zhang, Prof. B. Z. Tang
Department of Polymer Science and Engineering, Zhejiang University, Xihu District, Hangzhou 310027, China
Email: zhanghaoke@zju.edu.cn

X. Wu, X. Chen, Prof. A. Qin, Prof. B. Z. Tang
Center for Aggregation-Induced Emission, SCUT-HKUST Joint Research Institute, State Key Laboratory of Luminescent Materials and Devices, South China University of Technology, Guangzhou 510640, China

Prof. B. Z. Tang
AIE Institute, Guangzhou Development District, Huangpu, Guangzhou 510530, China

Table of contents

**Materials and Methods**	S3
**Synthesis and Characterizations**	S4
**Scheme S1**. Synthetic route to the compounds.	S4
**Table S1**. Crystal Data and Structure Refinement of ETMI, DETMI and DETSI	S19
**Table S2.** Optical properties and energy levels of luminogens	S20
**Table S3.** Major electronic excitations in MI, ETMI, DETMI, SI, ETSI and DETSI determined by the TD-DFT method.	S20
**Figure S25.** Hydrodynamic radius distribution of MI at different concentrations.	S21
**Figure S26**. UV/vis absorption of MI in MeCN with different concentration.	S21
**Figure S27.** The distance between two sulfur atoms of DETMI showing strong S・・・S interaction.	S21
**Figure S28**. UV/vis absorption of SI, ETSI and DETSI, calculated by using B3LYP/6-31G*/CPCM/MeCN basis set with G03 program.	S22
**Figure S29.** Molecules structures, molecular orbital and energy levels of HOMOs and LUMOs of SI, ETSI and DETSI, calculated by using B3LYP/6-31G(d,p) basis set with G03 program. (Purple: nitrogen atom; yellow: sulfur atom; red: oxygen atom; cyan: carbon atom; white: hydrogen atom).	S22
**References**	S23

Materials and Methods

Materials. Maleimide (MI) succinimide (SI), and sodium acetate trihydrate were purchased from Meryer. Ethanethiol **2** was purchased from Aldrich. 2,3-Dibromomaleinimide **3** was purchased from Energy. Bromomaleimide **1** was prepared in the laboratory according to (modified) literature method.[1] All the commercially available reactants and reagents were used as received without further purification.

Instruments. All final products were purified by Versatile Preparative HPLC System (YMC LC-Forte/R) and used a high-performance liquid chromatograph (Waters e2695) for analysis. ^{1}H and ^{13}C NMR spectra were obtained on a Bruker ARX 400 NMR spectrometer in CDCl3 using tetramethylsilane (TMS, δ = 0) as internal reference. Photoluminescence (PL) spectra were measured on a PerkinElmer LS 55 spectrophotometer. UV-vis absorption spectra were measured on a Shimadzu UV-2600 spectrophotometer. Quantum yields were measured using a Hamamatsu absolute PL quantum yield spectrometer C11347 Quantaurus_QY. High resolution mass spectra (HRMS) were performed on a GCT Premier CAB 048 mass spectrometer.

Computational Details

DFT calculations were performed with the Gaussian 09 program,[2] at the B3LYP/6-31G* levels of theory. Geometry optimizations were performed without any symmetry constraints, and frequency calculations on the resulting optimized geometries showed no imaginary frequencies. Electronic transitions were calculated by the time-dependent DFT (TD-DFT) method. The MO contributions were generated using the Multiwfn package and plotted using GaussView 5.0. The solvation effects in dichloromethane are included for a part of the calculations with the conductor-like polarizable continuum model (CPCM).[3]

Synthesis and Characterizations

MI and SI are commercially available and were purchased from Meryer. The other four target compounds ETMI, DETMI, ETSI and DETSI were prepared by the synthetic route presented in Scheme S1.

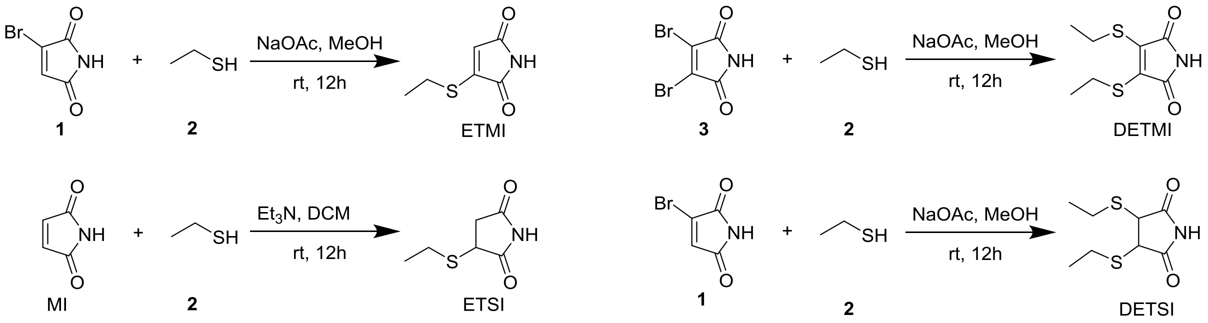

Scheme S1. Synthetic routes to the compounds.

Synthesis of compound ETMI: Into a 100 mL two-necked round bottom flask, the bromomaleimide **1** (1.05 g, 6 mmol) and sodium acetate trihydrate (898 mg, 6.6 mmol) were dissolved in 25 mL methanol. Then 450 μL ethanethiol (373 mg, 6 mmol) was added and stirred at room temperature for 12h. After reaction, the mixture was extracted with dichloromethane three times. The organic layer was collected and dried over anhydrous magnesium sulfate. After solvent evaporation, the crude product was first purified by silica gel column chromatography (hexane: ethyl acetate = 4:1) and then purified by Versatile Preparative HPLC System (YMC LC-Forte/R, condition: YMC-Pach SIL (250 × 20.0 mm I.D. column; 5 μm), mobile phase hexane: ethyl acetate = 80:20 at 7.0 ml/min with UV detection at 254 nm and 210 nm). Light yellow solid of ETMI was isolated in 70% yield. ^{1}H NMR (400 MHz, $CDCl_3$), δ (ppm): 7.65 (s, NH, 1H), 6.05 (s, =CH, 1H), 2.94 (t, CH_2, J = 7.28, 2H), 1.43 (t, CH_3, J = 7.12 Hz, 3H). ^{13}C NMR (100 MHz, $CDCl_3$), δ (ppm): 169.38, 167.93, 152.49, 118.40, 26.22, 12.99. HRMS ($C_6H_7NO_2S$): *m/z* 158.0275 (M + H+, calcd 158.0270).

Synthesis of compound DETMI: Into a 100 mL two-necked round bottom flask, the 2,3-Dibromomaleinimide **3** (1.27 g, 5 mmol) and sodium acetate trihydrate (1.5 g, 11 mmol) were dissolved in 30 mL methanol. Then 815 μL ethanethiol (683 mg, 11 mmol) was added and

stirred at room temperature for 12h. After reaction, the mixture was extracted with dichloromethane three times. The organic layer was collected and dried over anhydrous magnesium sulfate. After solvent evaporation, the crude product was first purified by silica gel column chromatography (hexane: ethyl acetate = 9:1) and then purified by Versatile Preparative HPLC System (YMC LC-Forte/R; condition: YMC-Pach SIL (250 × 20.0 mm I.D. column; 5 μm), mobile phase hexane: ethyl acetate = 90:10 at 7.0 ml/min with UV detection at 254 nm and 210 nm). Yellow solid of DETMI was isolated in 85% yield. ^{1}H NMR (400 MHz, $CDCl_3$), *δ* (ppm): 7.82 (s, NH, 1H), 3,31 (q, CH_2, *J* = 7.36, 4H), 1.33 (t, CH_3, *J* = 7.40 Hz, 3H). ^{13}C NMR (100 MHz, $CDCl_3$), *δ* (ppm): 166.50, 136.62, 26.38, 15.68. HRMS ($C_8H_{11}NO_2S_2$): *m/z* 218.0311 (M + H+, calcd 218.0304).

Synthesis of compound ETSI: Into a 100 mL two-necked round bottom flask, the maleimide (1.45 mg, 15 mmol) was dissolved in 30 mL dichloromethane. Then 1.33 mL ethanethiol (1.12 g, 18 mmol) and 250 μL triethylamine (182 mg, 1.8 mmol) were added and stirred at room temperature for 12h. After reaction, the mixture was extracted with dichloromethane three times. The organic layer was collected and dried over anhydrous magnesium sulfate. After solvent evaporation, the crude product was first purified by silica gel column chromatography (hexane: ethyl acetate = 3:2) and then purified by Versatile Preparative HPLC System (YMC LC-Forte/R; condition: YMC-Pach SIL (250 × 20.0 mm I.D. column; 5 μm), mobile phase hexane: ethyl acetate = 60:40 at 7.0 ml/min with UV detection at 254 nm and 210 nm). Light yellow viscous of ETSI was isolated in 80% yield. ^{1}H NMR (400 MHz, $CDCl_3$), *δ* (ppm): 8.33 (s, NH, 1H), 3.77 (m, 1H), 3.21 (m, 1H), 2.91 (m, 1H), 2.80 (m, 1H), 2.56 (dd, *J* = 3.84 Hz, 1H), 1.32 (t, CH_3, *J* = 7.40 Hz, 3H). ^{13}C NMR (100 MHz, $CDCl_3$), *δ* (ppm): 176.79, 174.89, 40.22, 37.39, 26.05, 14.23. HRMS ($C_6H_9NO_2S$): *m/z* 160.0436 (M + H+, calcd 160.0427).

Synthesis of compound DETSI: Into a 100 mL two-necked round bottom flask, the bromomaleimide **1** (1.75 g, 10 mmol) and sodium acetate trihydrate (1.63 g, 12 mmol) were

dissolved in 40 mL methanol. Then 1.63 mL ethanethiol (1.37 g, 22 mmol) was added and stirred at room temperature for 12h. After reaction, the mixture was extracted with dichloromethane three times. The organic layer was collected and dried over anhydrous magnesium sulfate. After solvent evaporation, the crude product was first purified by silica gel column chromatography (hexane: ethyl acetate = 4:1) and then purified by Versatile Preparative HPLC System (YMC LC-Forte/R; condition: YMC-Pach SIL (250 × 20.0 mm I.D. column; 5 μm), mobile phase hexane: ethyl acetate = 85:15 at 7.0 ml/min with UV detection at 254 nm and 210 nm). Colorless solid of DETSI was isolated in 78% yield. ^{1}H NMR (400 MHz, $CDCl_3$), δ (ppm): 8.77 (s, NH, 1H), 3,52 (s, CH, 2H), 2.94-2.75 (m, 4H), 1.32 (t, CH_3, J = 7.40 Hz, 6H). ^{13}C NMR (100 MHz, $CDCl_3$), δ (ppm): 175.06, 47.92, 26.55, 14.27. HRMS ($C_8H_{13}NO_2S_2$): *m/z* 220.0444 (M + H+, calcd 220.0460).

Structural Characterization

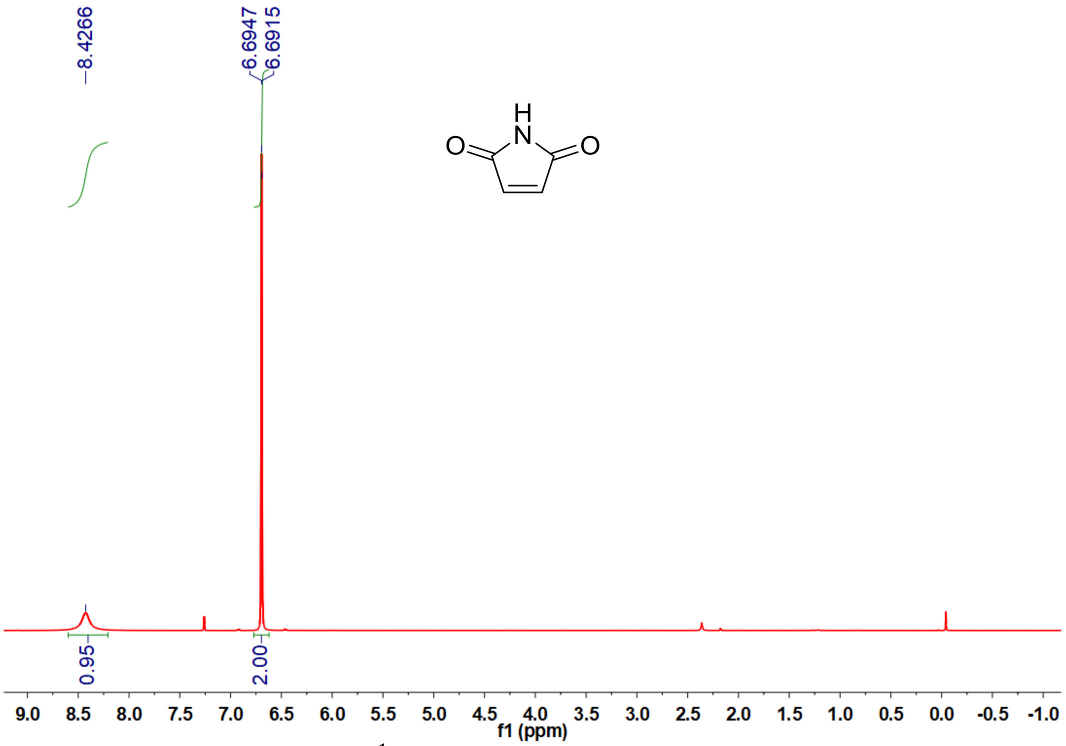

Figure S1. ^{1}H NMR spectrum of MI in $CDCl_3$.

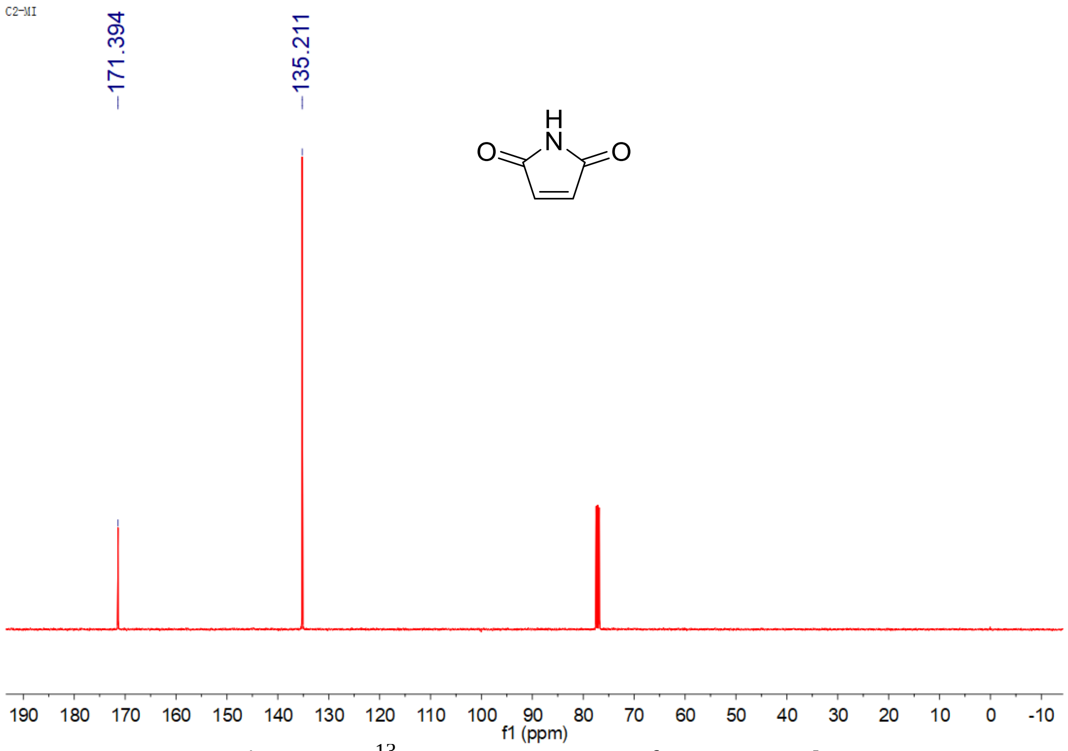

Figure S2. ^{13}C NMR spectrum of MI in $CDCl_3$.

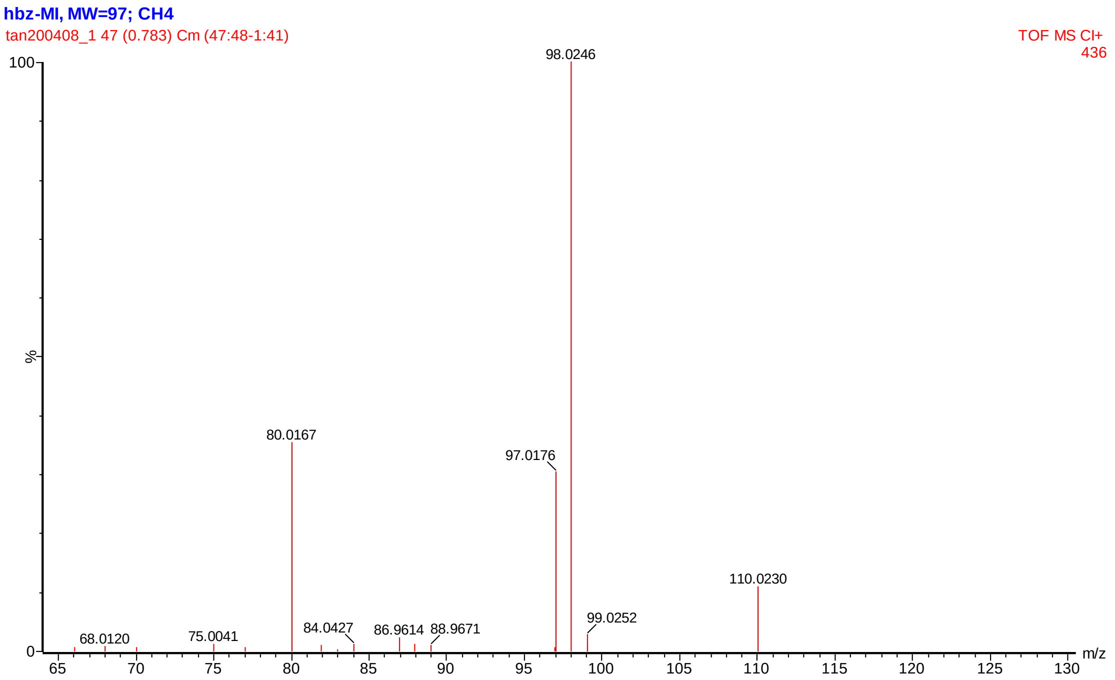

Figure S3. HR-MS spectrum of MI in THF.

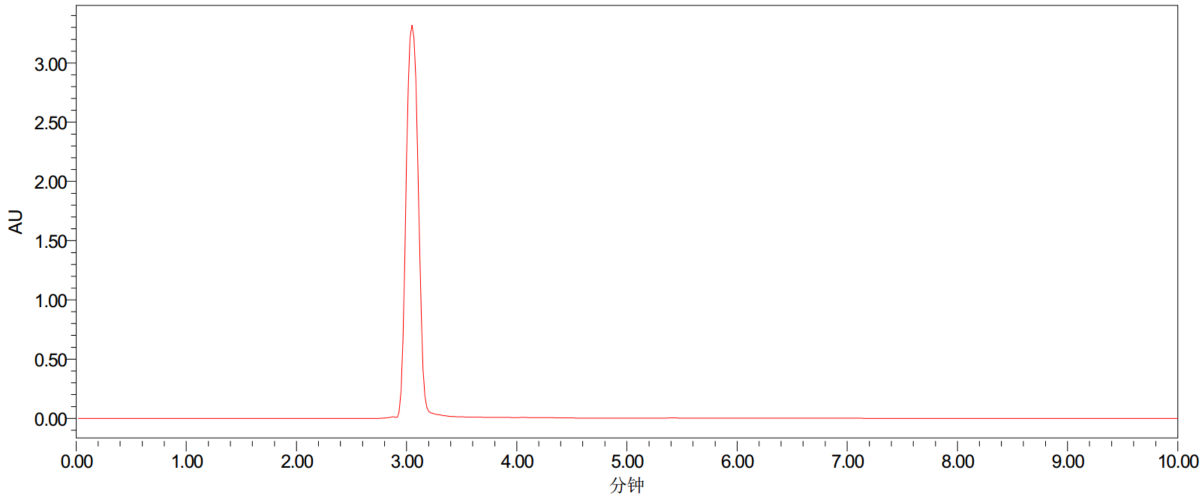

Figure S4. Reverse-phase HPLC analysis spectra of MI. Condition: SunFire C18 (4.6 × 250 mm column; 5 μm), mobile phase acetonitrile at 1.0 ml/min with UV detection at 254 nm (room temperature).

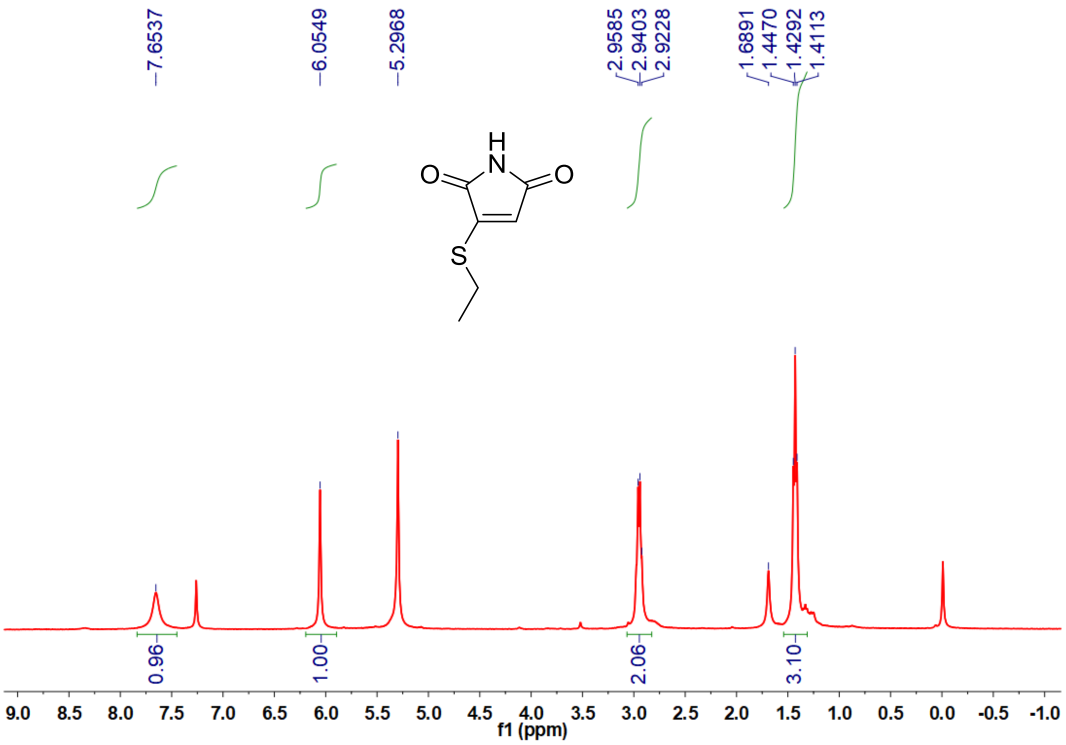

Figure S5. ^{1}H NMR spectrum of ETMI in $CDCl_3$.

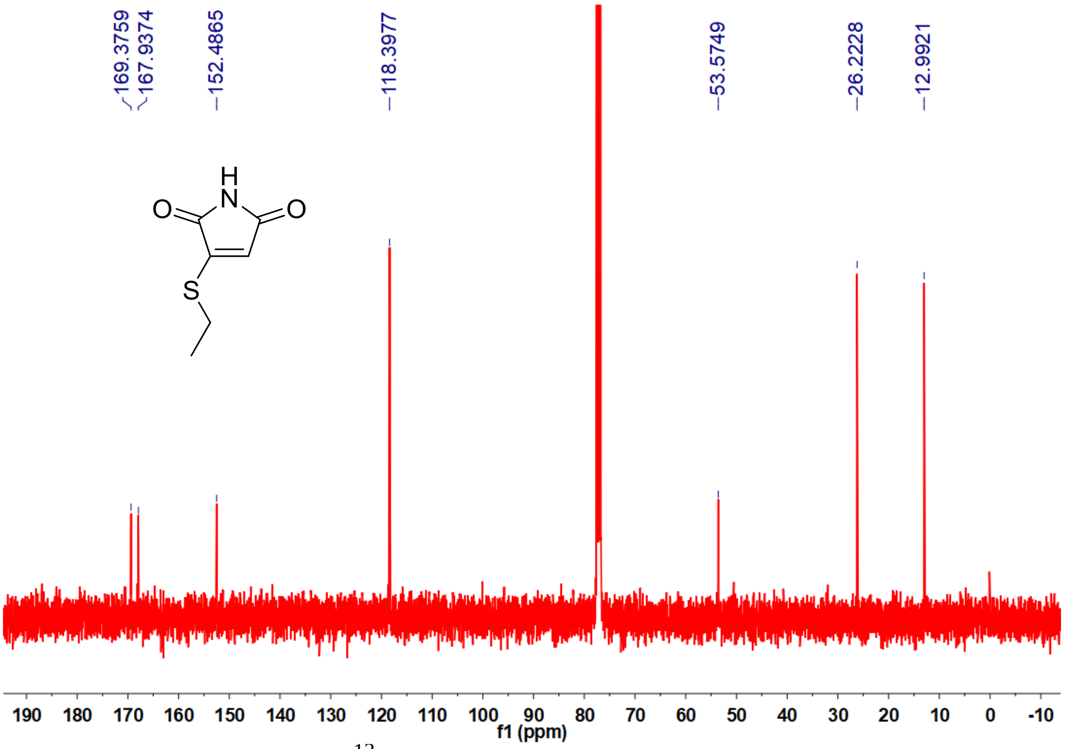

Figure S6. ^{13}C NMR spectrum of ETMI in $CDCl_3$.

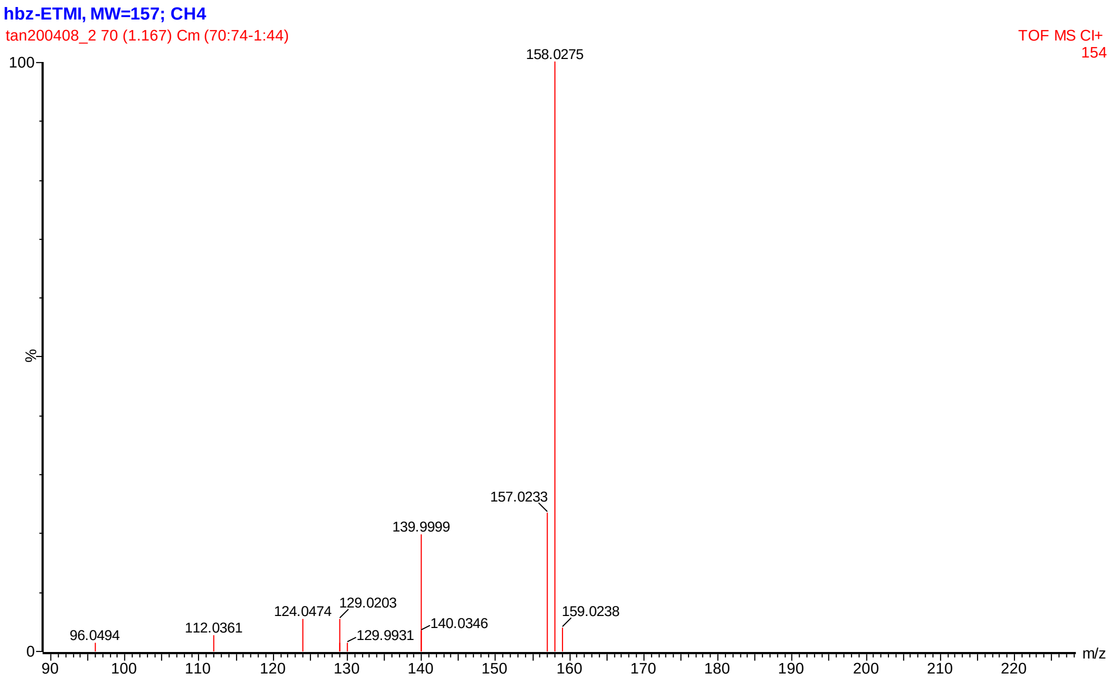

Figure S7. HR-MS spectrum of ETMI in THF.

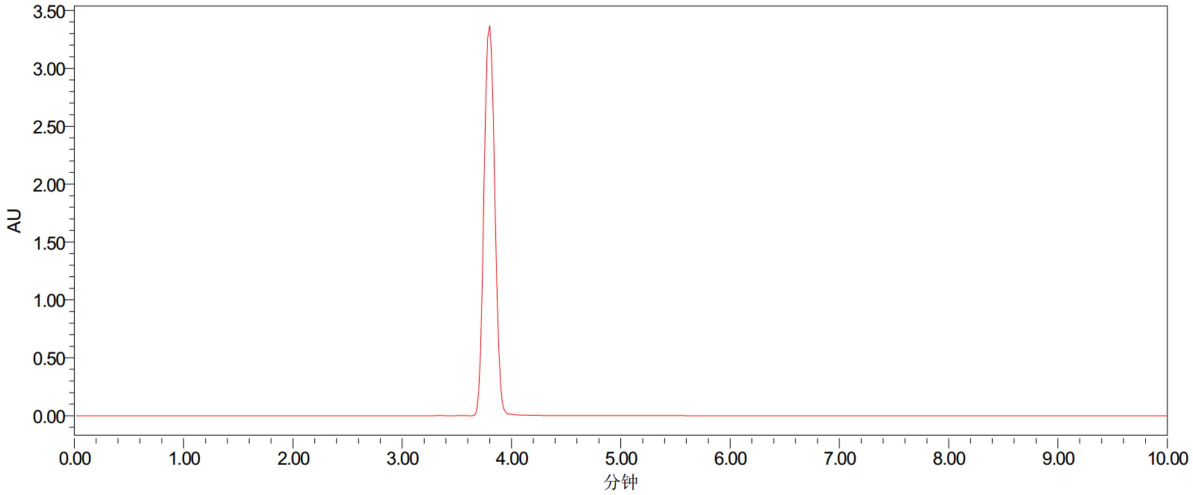

Figure S8. Reverse-phase HPLC analysis spectra of ETMI. Condition: SunFire C18 (4.6 × 250 mm column; 5 µm), mobile phase acetonitrile at 1.0 ml/min with UV detection at 254 nm (room temperature).

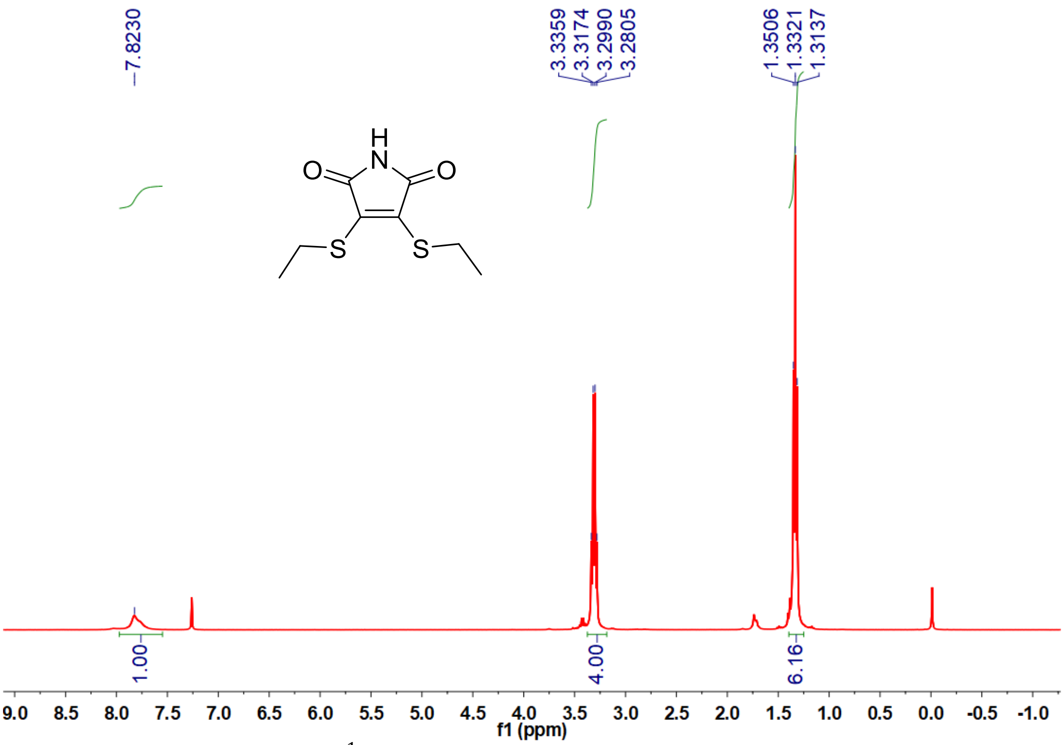

Figure S9. ^{1}H NMR spectrum of DETMI in $CDCl_3$.

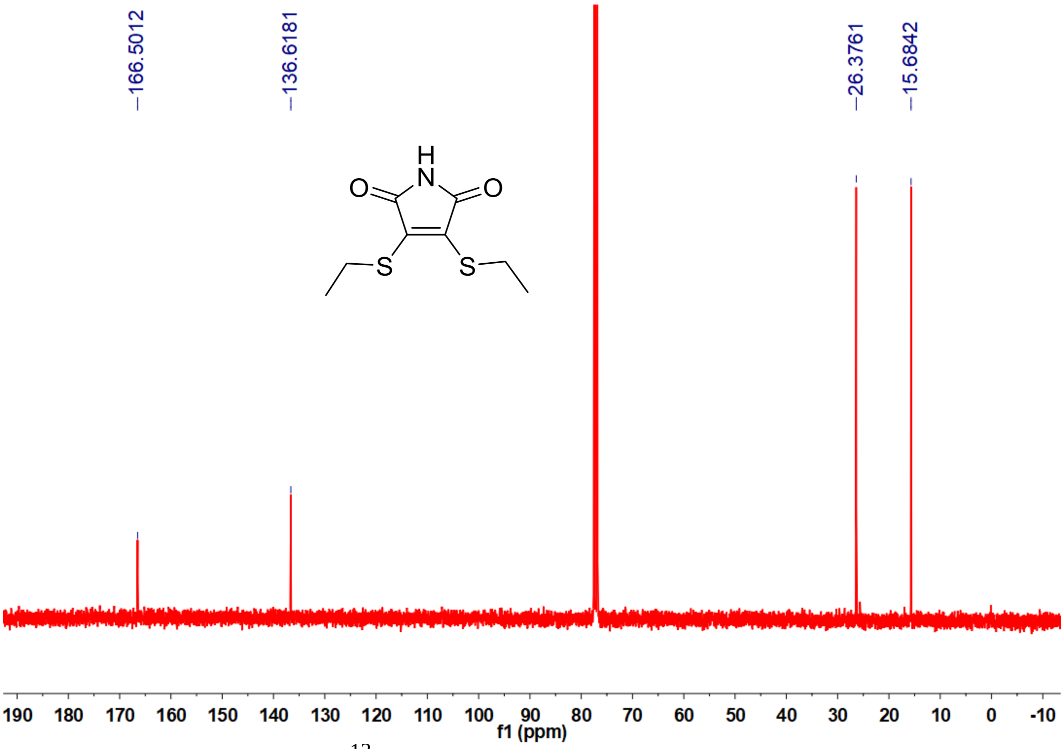

Figure S10. ^{13}C NMR spectrum of DETMI in $CDCl_3$.

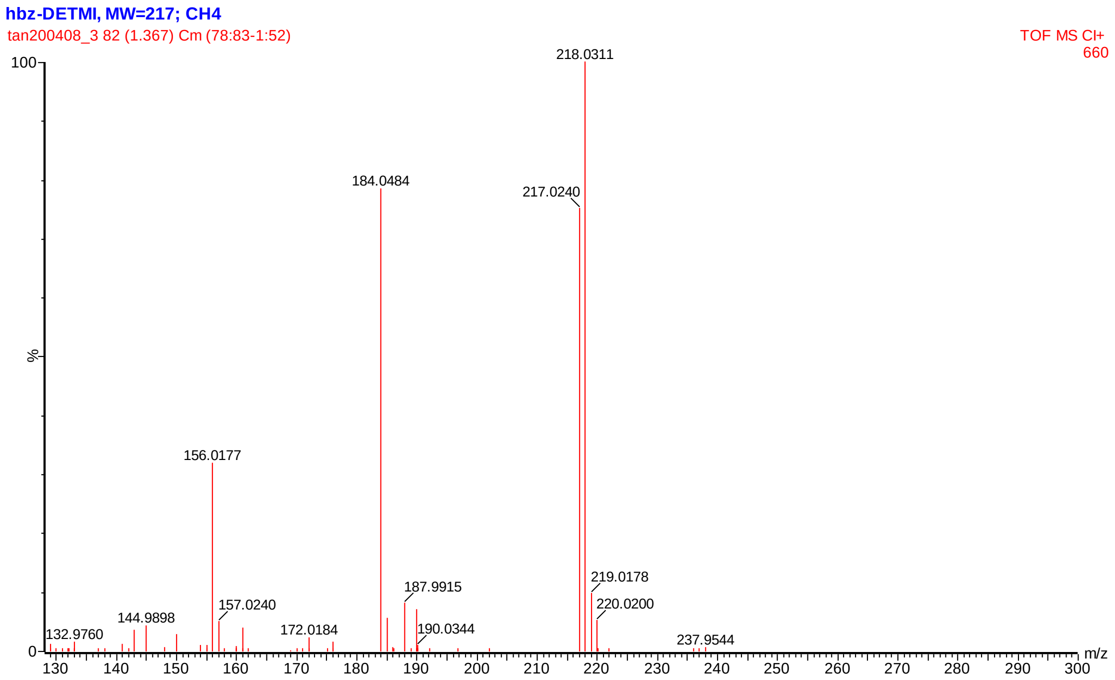

Figure S11. HR-MS spectrum of DETMI in THF.

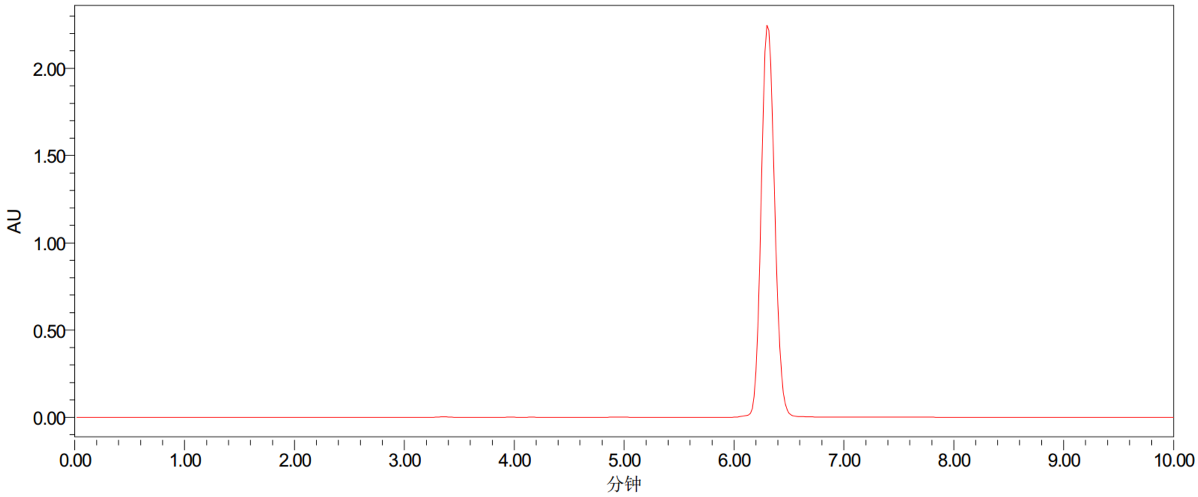

Figure S12. Reverse-phase HPLC analysis spectra of DETMI. Condition: SunFire C18 (4.6 × 250 mm column; 5 μm), mobile phase acetonitrile at 1.0 ml/min with UV detection at 254 nm (room temperature).

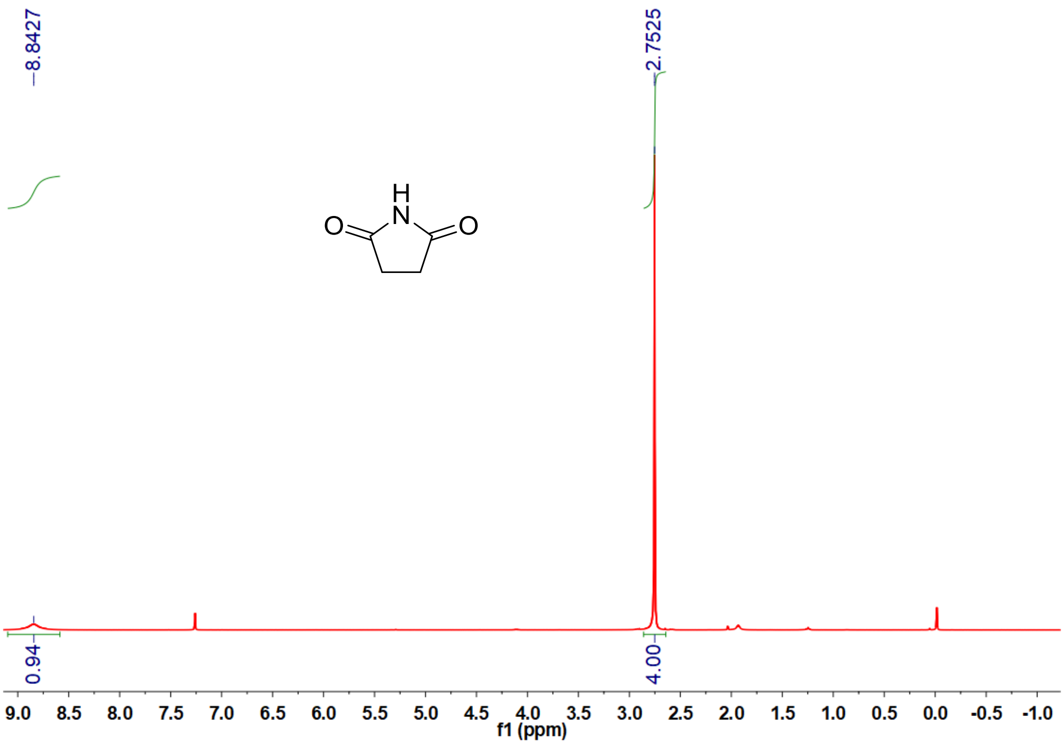

Figure S13. ^{1}H NMR spectrum of SI in $CDCl_3$.

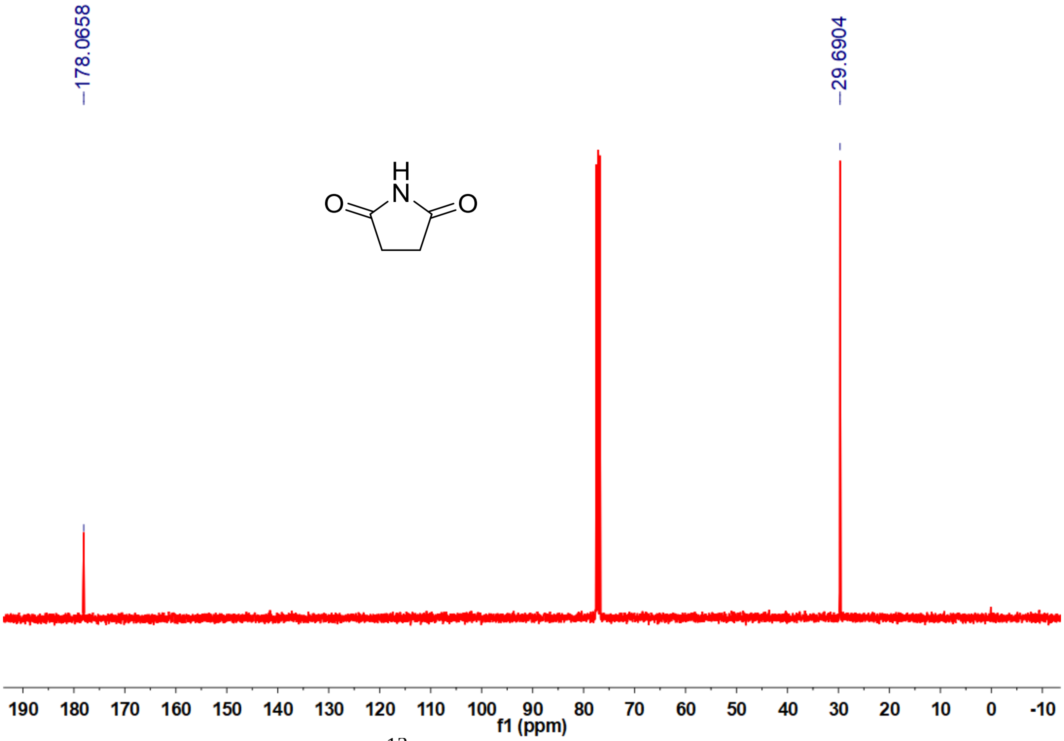

Figure S14. ^{13}C NMR spectrum of SI in $CDCl_3$.

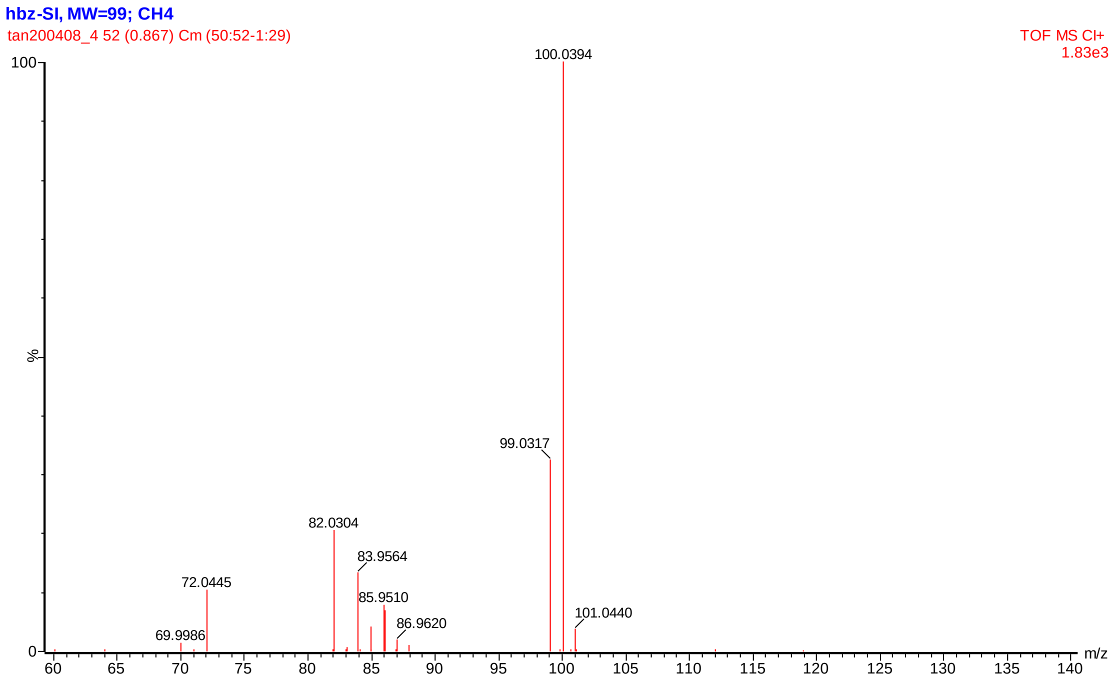

Figure S15. HR-MS spectrum of SI in THF.

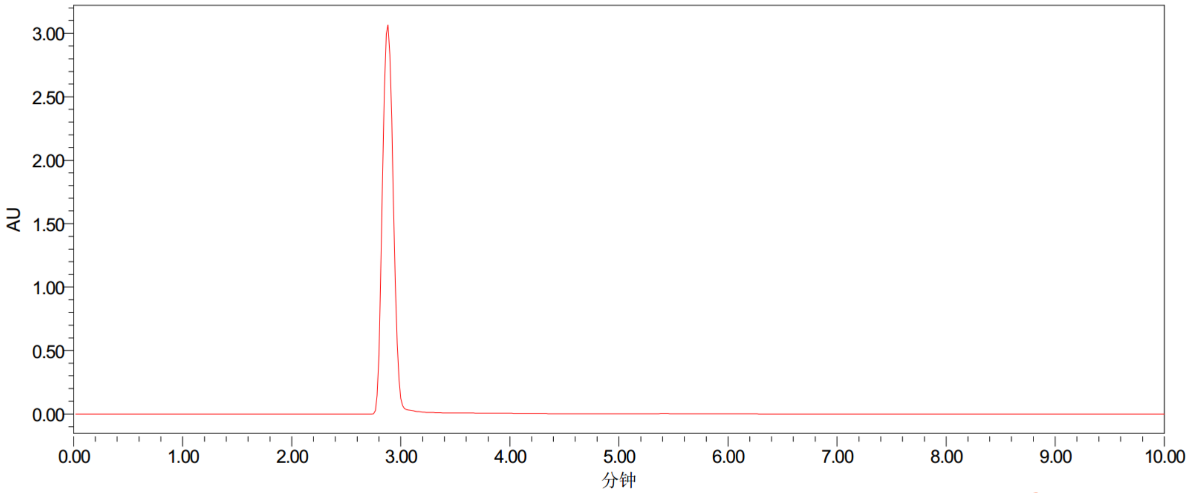

Figure S16. Reverse-phase HPLC analysis spectra of SI. Condition: SunFire C18 (4.6 × 250 mm column; 5 μm), mobile phase acetonitrile at 1.0 ml/min with UV detection at 254 nm (room temperature).

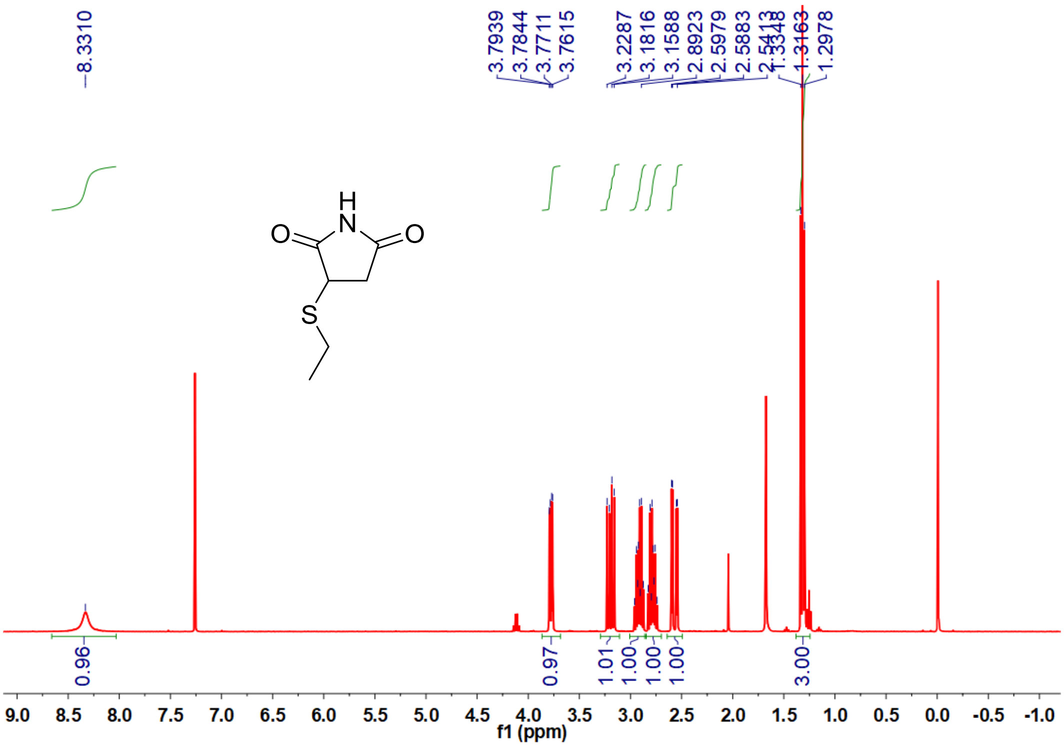

Figure S17. ¹H NMR spectrum of ETSI in $CDCl_3$.

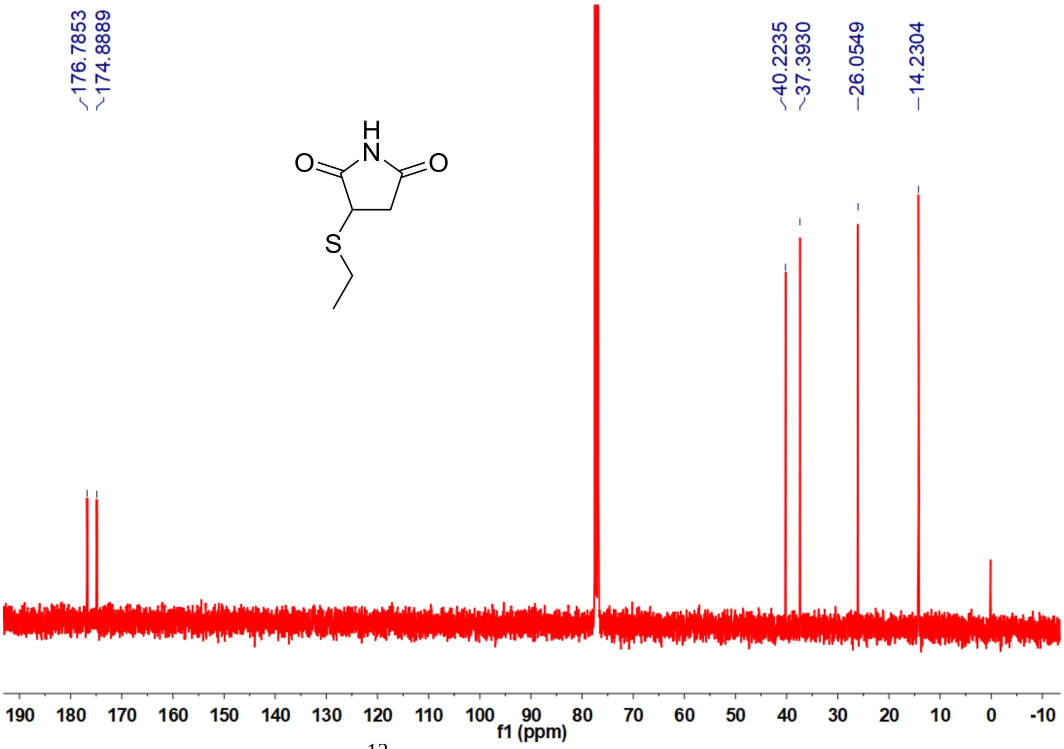

Figure S18. ¹³C NMR spectrum of ETSI in $CDCl_3$.

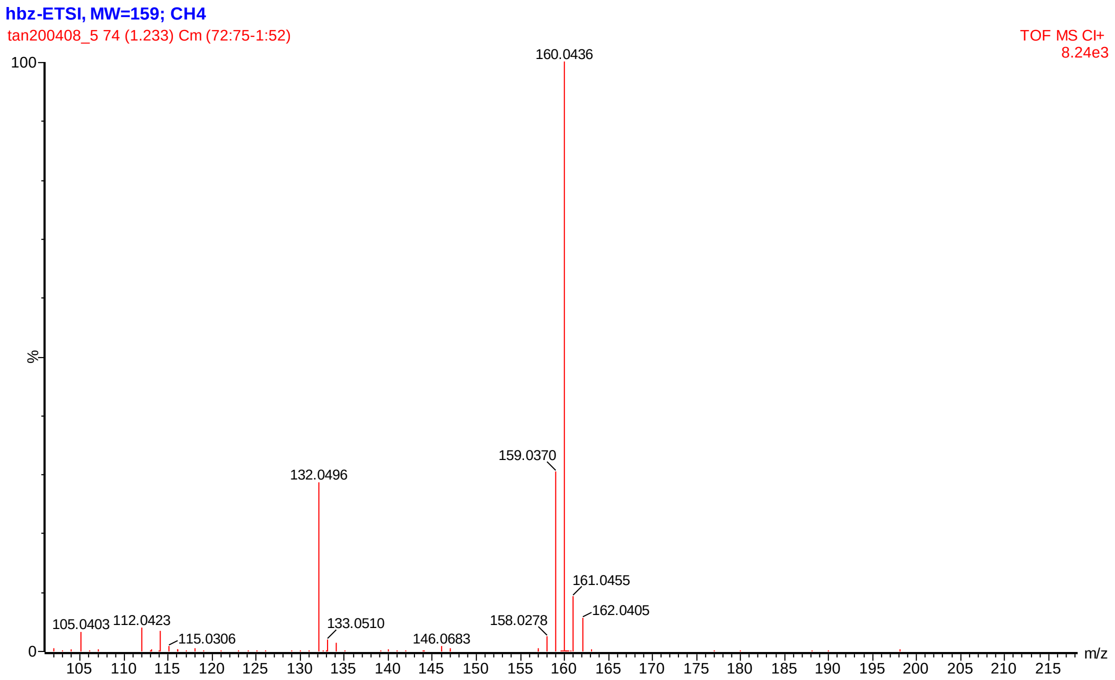

Figure S19. HR-MS spectrum of ETSI in THF.

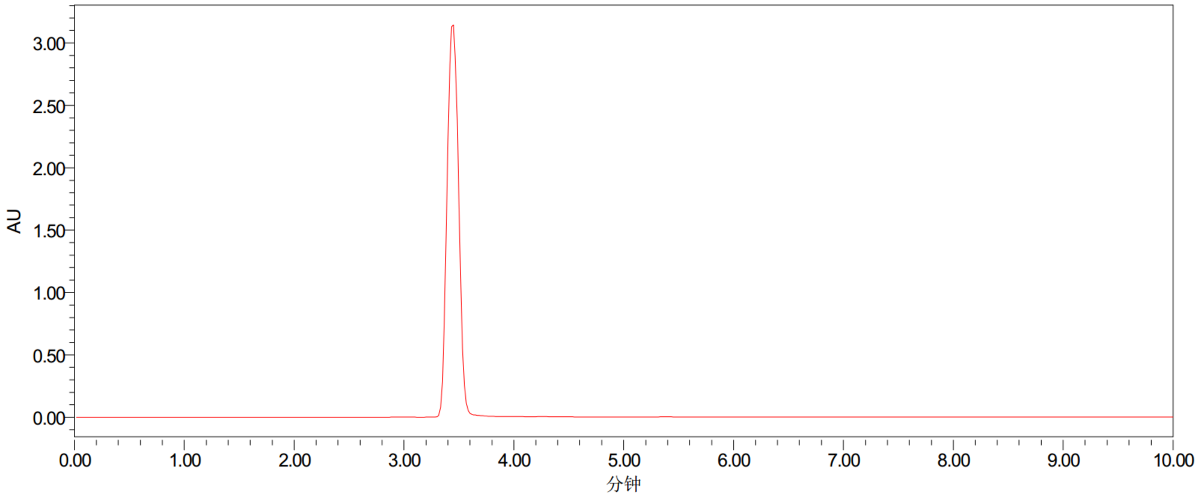

Figure S20. Reverse-phase HPLC analysis spectra of ETSI. Condition: SunFire C18 (4.6 × 250 mm column; 5 μm), mobile phase acetonitrile at 1.0 ml/min with UV detection at 254 nm (room temperature).

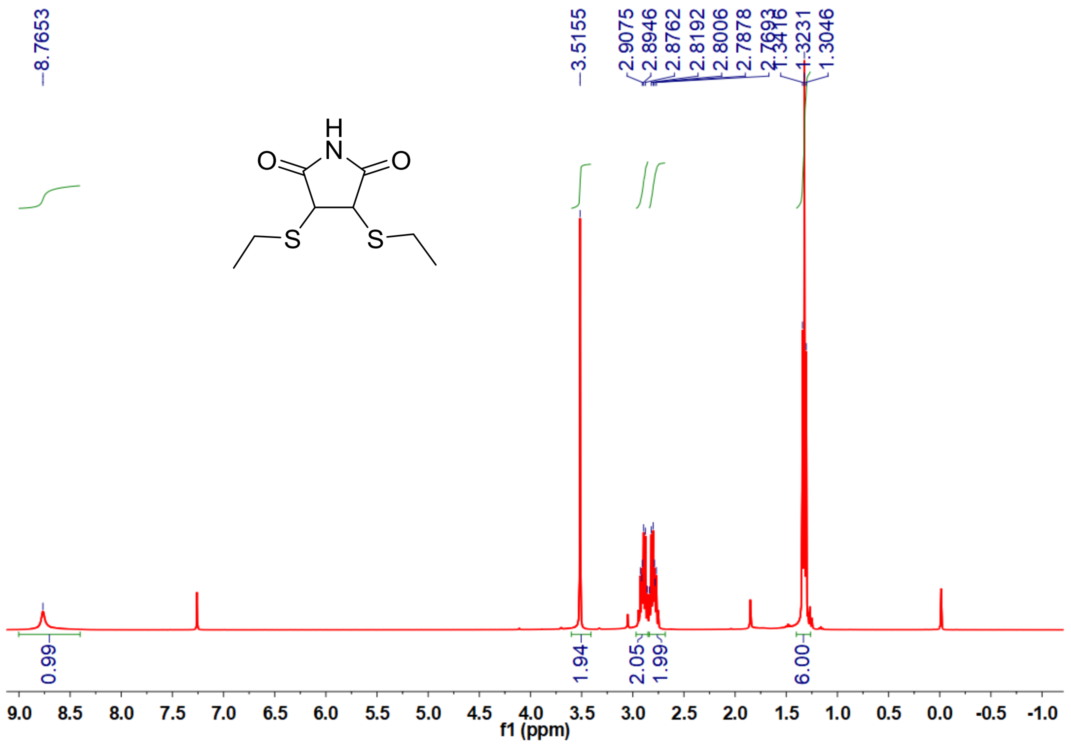

Figure S21. ^{1}H NMR spectrum of DETSI in $CDCl_3$.

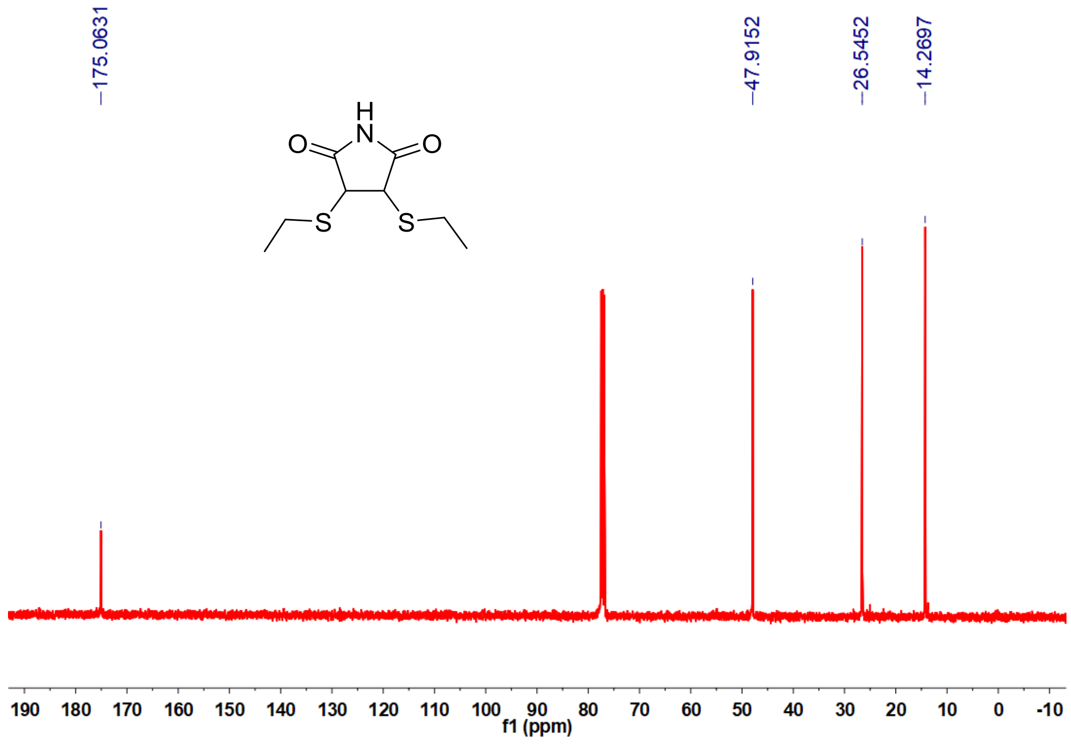

Figure S22. ^{13}C NMR spectrum of DETSI in $CDCl_3$.

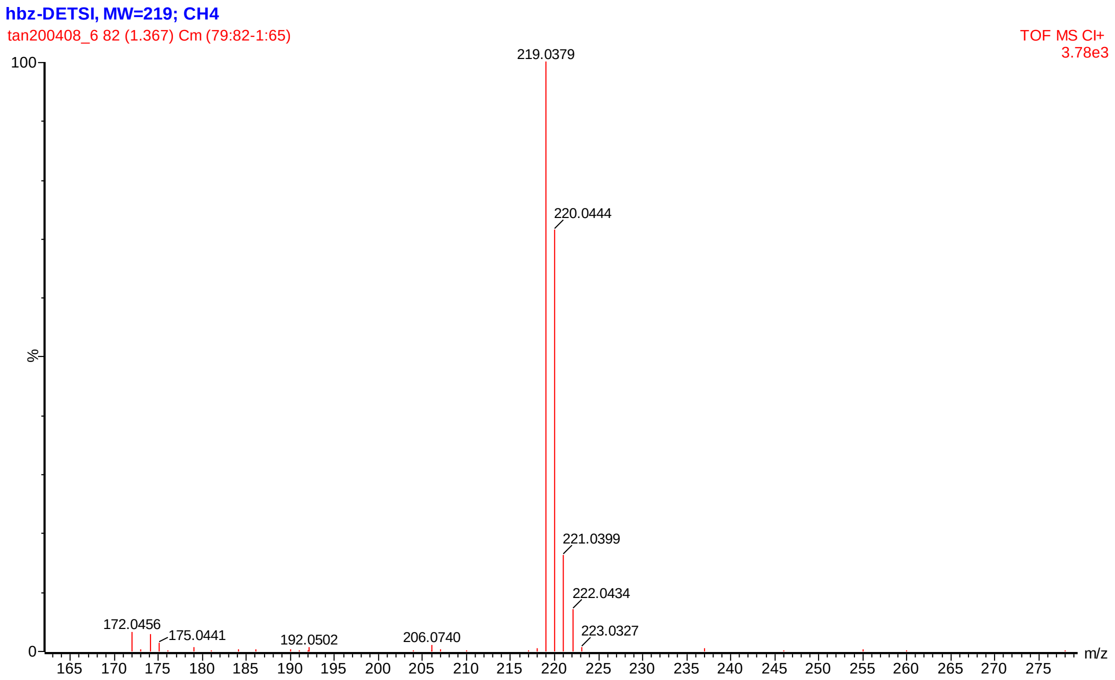

Figure S23. HR-MS spectrum of DETSI in THF.

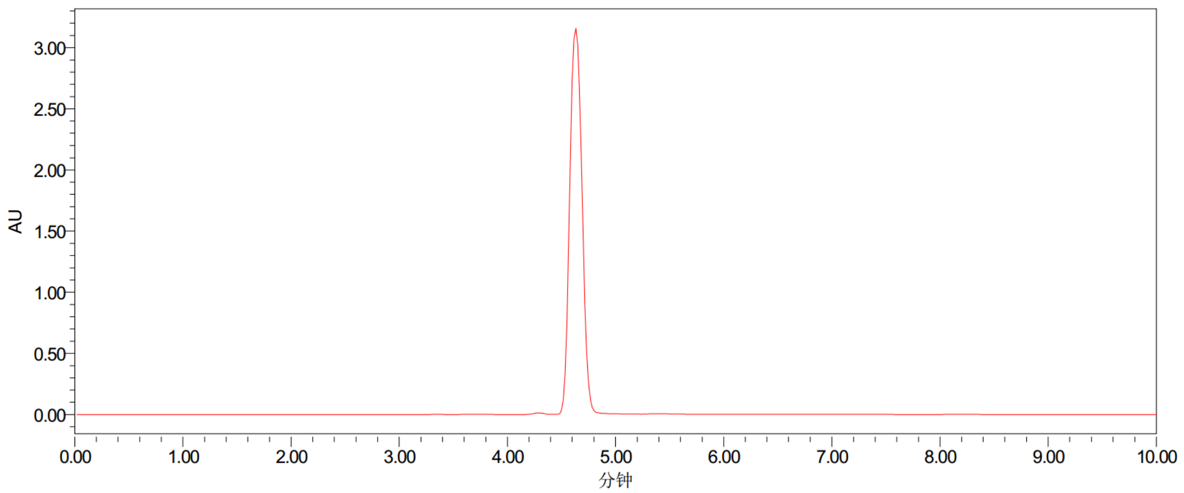

Figure S24. Reverse-phase HPLC analysis spectra of DETSI. Condition: SunFire C18 (4.6 × 250 mm column; 5 μm), mobile phase acetonitrile at 1.0 ml/min with UV detection at 254 nm (room temperature).

Table S1. Crystal Data and Structure Refinement of ETMI, DETMI and DETSI.[a]

Identification code	ETMI	DETMI	DETSI
Empirical formula	$C_6H_7NO_2S$	$C_8H_{11}NO_2S_2$	$C_8H_{13}NO_2S_2$
Formula weight	157.19	217.30	219.31
Temperature (K)	100.00(10)	100.00(10)	100.00(10)
Crystal system	monoclinic	monoclinic	monoclinic
Space group	$P2_1$/n	$P2_1$/n	*C*2/c
a (Å)	5.23122(10)	5.39356(16)	20.7884(5)
b (Å)	8.03781(15)	8.6151(3)	12.1671(3)
c (Å)	16.1471(3)	21.8761(7)	8.4491(2)
α (°)	90	90	90
β (°)	92.6319(16)	93.052(3)	100.258(2)
γ (°)	90	90	90
Volume ($Å^3$)	678.23(2)	1015.05(5)	2102.91(9)
Z	4	4	8
ρ_{calc}g (cm^3)	1.539	1.422	1.385
μ (mm^{-1})	3.713	4.511	4.355
F(000)	328.0	456.0	928.0
Crystal size (mm^3)	0.12 × 0.1 × 0.1	0.2 × 0.1 × 0.05	0.2 × 0.2 × 0.03
Radiation	CuKα (λ = 1.54184)	CuKα (λ = 1.54184)	CuKα (λ = 1.54184)
2Θ range for data collection (°)	10.97 to 134.912	8.094 to 147.58	8.456 to 147.372
Index ranges	$-6 \le h \le 4$, $-9 \le k \le 9$, $-19 \le l \le 16$	$-4 \le h \le 6$, $-8 \le k \le 10$, $-26 \le l \le 26$	$-25 \le h \le 24$, $-14 \le k \le 7$, $-10 \le l \le 10$
Reflections collected	3447	5331	5845
Independent reflections	1226 [R_{int} = 0.0156, R_{sigma} = 0.0161]	1986 [R_{int} = 0.0360, R_{sigma} = 0.0352]	2085 [R_{int} = 0.0274, R_{sigma} = 0.0257]
Data/restraints/parameters	1226/0/92	1986/0/120	2085/0/120
Goodness-of-fit on F^2	1.009	1.102	1.029
Final R indexes [I>=2σ (I)]	R_1 = 0.0238, wR_2 =0.0635	R_1 = 0.0587, wR_2 = 0.1496	R_1 = 0.0410, wR_2 = 0.1131
Final R indexes [all data]	R_1 = 0.0247, wR_2 = 0.0639	R_1 = 0.0612, wR_2 = 0.1507	R_1 = 0.0423, wR_2 = 0.1161
Largest diff. peak/hole (e $Å^{-3}$)	0.31/-0.24	0.90/-0.35	0.85/-0.45

[a] Crystallographic data for the structures reported in this work have been deposited with the Cambridge Crystallographic Data Centre as supplementary publication no. CCDC: 2032995 for EMI, 2033012 for DETMI, and 2033020 for DETSI. Note: MI and SI are known, their cif files were downloaded from CCDC, and their CCDC numbers are: 651626 and 1263491, respectively.

Table S2. Optical properties and energy levels of luminogens

	λ_{ab} (nm)	ε (L mol^{-1} cm^{-1})	λ_{ex} (nm)		λ_{em} (nm)		Φ_F (%)		CCC (mol/L)	Proportions of transition^{f}
			Soln^{a}	Solid	Soln^{b}	Solid	Soln^{c}	Solid		
MI	271	840	354	396	456	475	0.3	3.6	1.4×10^{-3}	$\alpha_n >> \beta_\pi$
ETMI	340	4990	400	446	500	529	1.0	1.8	1.7×10^{-3}	$\alpha_n > \beta_\pi$
DETMI	395	4710	410	465	513^{c}	540	0.8	17.7	1.9×10^{-5}	$\alpha_n << \beta_\pi$
SI	242	120	353	375	465	480	0.1	0.7	3.2×10^{-3}	$\alpha_n >> \beta_\pi$
ETSI	263	360	360	400^{e}	489	513^{e}	0.1	0.9^{e}	2.1×10^{-3}	$\alpha_n >> \beta_\pi$
DETSI	338	280	364	410	491	511	0.1	2.6	2.2×10^{-3}	$\alpha_n >> \beta_\pi$

^{a} Solution concentration is 100 μM. ^{b} Solution concentration is 10 mM. ^{c} Solution concentration is 10 μM. ^{d} Solution concentration of DETMI is 100 μM. ^{e} Sample is liquid at room temperature. ^{f} The proportions of the (n,π*) and (π,π*) transitions are defined as α_n and β_π, respectively, with $\alpha_n + \beta_\pi = 1$. Abbreviation: Soln = solution, ε = molar extinction coefficient, CCC = critical cluster concentration.

Table S3. Major electronic excitations in MI, ETMI, DETMI, SI, ETSI and DETSI determined by the TD-DFT method.^{a}

Compound	Excited State	λ (nm) [Expt.]	Osc. Strength (f)	Major Contributions
MI	S_1	355 [not found]	0.0001	HOMO→LUMO, (99%)
	S_3	282 [271]	0.0015	HOMO-1→LUMO, (98%)
	S_4	217 [214]	0.3777	HOMO-3→LUMO, (95%)
ETMI	S_1	351 [340]	0.1166	HOMO→LUMO, (96%)
	S_4	245 [247]	0.1789	HOMO-2→LUMO, (80%)
	S_7	206 [193]	0.1532	HOMO-4→LUMO, (76%)
DETMI	S_1	432 [395]	0.1355	HOMO→LUMO, (98%)
	S_5	247 [226]	0.1705	HOMO-4→LUMO, (55%)<br>HOMO-3→LUMO, (22%)
	S_{13}	192 [195]	0.1770	HOMO-1→LUMO+1, (60%)<br>HOMO→LUMO+2, (28%)
SI	S_1	247 [273]	0.0012	HOMO→LUMO, (96%)
	S_4	177 [193]	0.3669	HOMO-2→LUMO, (96%)
ETSI	S_1	269 [263]	0.0016	HOMO→LUMO, (91%)
	S_7	182 [195]	0.2945	HOMO-3→LUMO, (73%)
DETSI	S_1	292 [264]	0.0064	HOMO-1→LUMO, (91%)
	S_{10}	189 [198]	0.3067	HOMO-4→LUMO, (84%)

^{a} The DFT method was B3LYP/6-31G(d,p)/CPCM/acetonitrile. S = singlet.

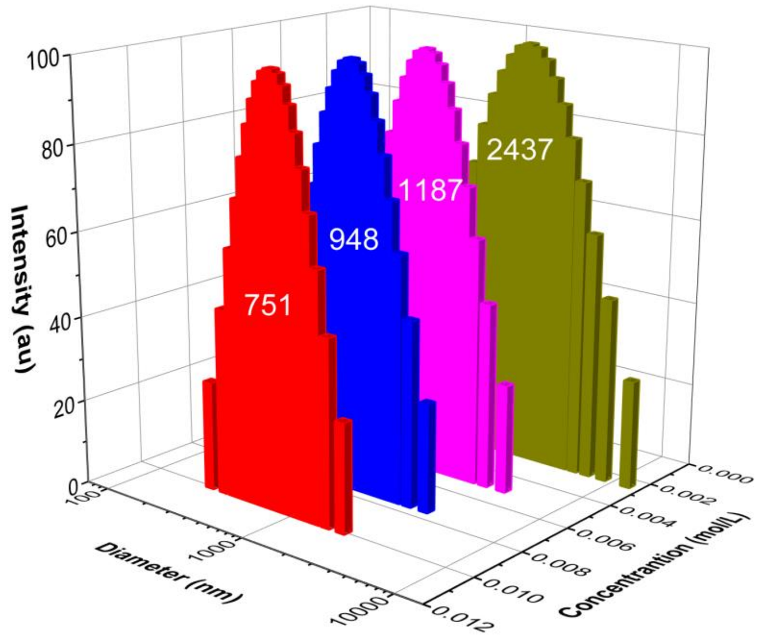

Figure S25. Hydrodynamic radius distribution of MI at different concentrations.

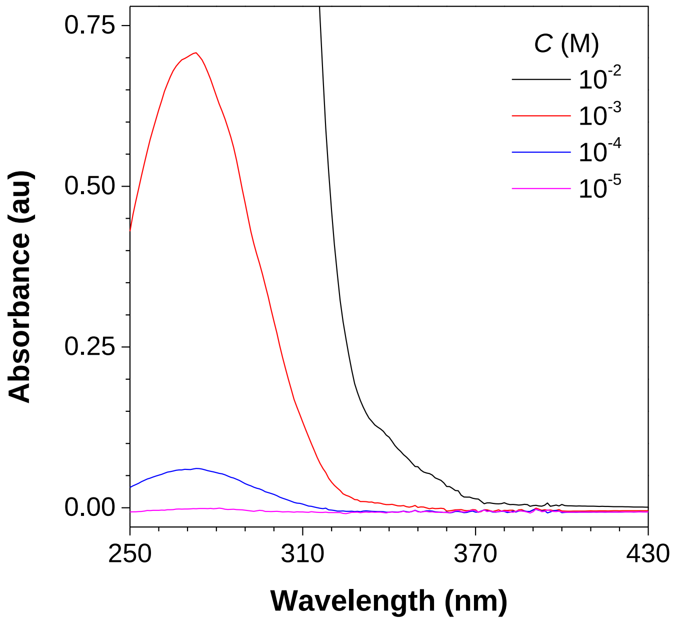

Figure S26. UV/vis absorption of MI in MeCN with different concentration.

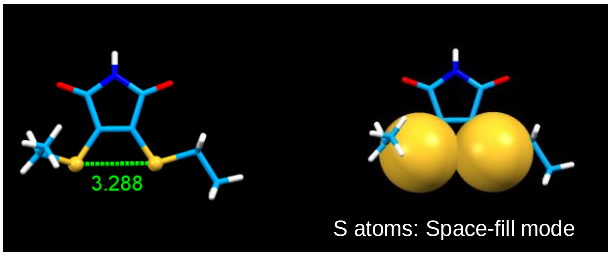

Figure S27. The distance between two sulfur atoms of DETMI showing strong S···S interaction.

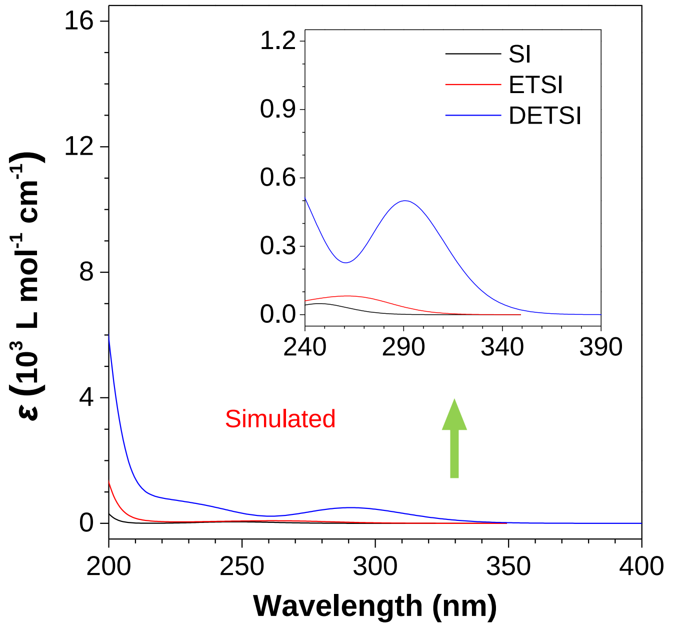

Figure S28. UV/vis absorption of SI, ETSI and DETSI, calculated by using B3LYP/6-31G*/CPCM/MeCN basis set with G03 program.

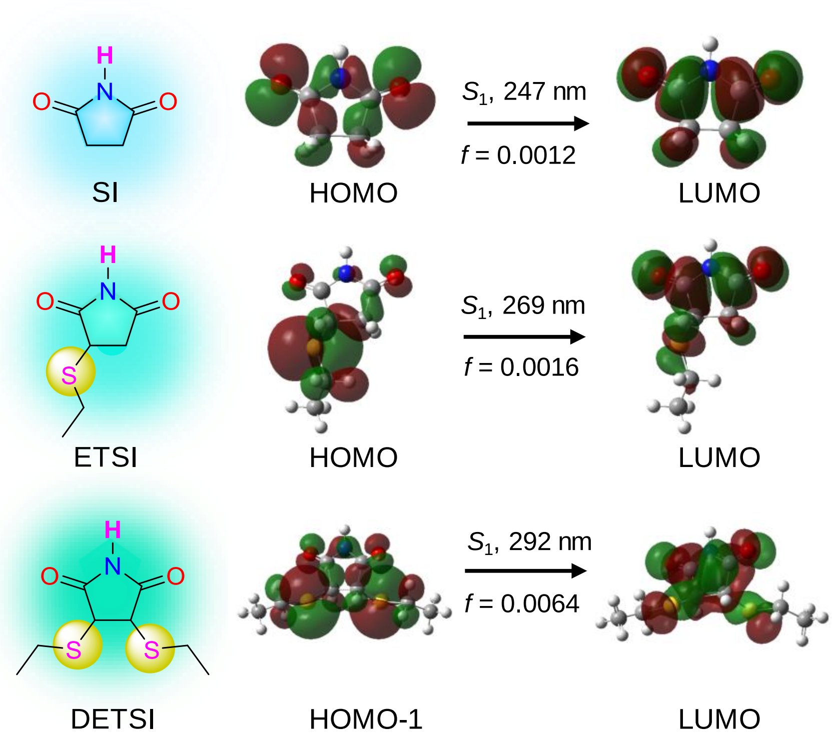

Figure S29. Molecules structures, molecular orbital and energy levels of HOMOs and LUMOs of SI, ETSI and DETSI, calculated by using B3LYP/6-31G(d,p) basis set with G03 program. (Purple: nitrogen atom; yellow: sulfur atom; red: oxygen atom; cyan: carbon atom; white: hydrogen atom).

References

[1] a) J. Yan, B. Zheng, D. Pan, R. Yang, Y. Xu, L. Wang, M. Yang, *Polym. Chem.* **2015**, *6*, 6133-6139; b) X. Li, H. Li, W. Yang, J. Zhuang, H. Li, W. Wang, *Tetrahedron Lett.* **2016**, *57*, 2660-2663.
[2] M. J. Frisch, G. W. Trucks, H. B. Schlegel, G. E. Scuseria, Gaussian, Inc., Wallingford CT, **2010**.
[3] V. Barone, M. Cossi, *J. Phys. Chem. A* **1998**, *102*, 1995-2001.
